# The experiential structure of dialect writing in new media: a mixed-methods study combining qualitative analysis and experimental perception evaluation

**DOI:** 10.3389/fpsyg.2026.1855166

**Published:** 2026-09-25

**Authors:** Qi Yang, Jing Lyu, Xiaojun Zhu

**Affiliations:** 1 School of Chinese Language and Literature, Xinjiang University, Urumqi, China; 2 Department of Foundational Education, Xinjiang Judicial Police Vocational College, Urumqi, China; 3 Urumqi No.149 Middle School (High-tech Zone Campus of No.1 Secondary School), Urumqi, China

**Keywords:** digital dialect, emotional contagion, exploratory factor analysis, social identity, social media, Xinjiang Chinese dialect

## Abstract

With the proliferation of social media and digital platforms, dialects are increasingly moving beyond traditional oral contexts into networked writing and digital interaction spaces. Compared to standard languages such as Mandarin, dialectal expressions on social media typically carry stronger emotional valence, interactivity, and regionally specific cultural features. However, existing research has mostly addressed digital dialect phenomena from sociolinguistic or cultural semiotic perspectives, and systematic empirical analyses of how users experientially perceive dialectal expressions and how these perceptual structures are formed remain scarce. Focusing on Xinjiang Chinese dialect expressions in social media, this study employs a combination of qualitative textual analysis and quantitative perception evaluation to examine the core experiential structures underlying digital dialect interactions. Drawing on dialect-interaction corpora from platforms such as Douyin and Bilibili, the study first conducts reflexive thematic analysis to identify key linguistic practices in digital dialect writing. Based on ten “dialect–Mandarin” stimulus pairs, an experimental perception evaluation was administered to 149 participants. The resulting perceptual patterns were examined through exploratory factor analysis (EFA), repeated measures analysis, correlation analysis, and demographic difference analyses. Results indicate that participants showed more favorable evaluations of Xinjiang Chinese dialect expressions compared to their Mandarin counterparts, particularly in terms of emotional contagion, vividness, and interactive proximity. Exploratory factor analysis further revealed that participants' perceptions of digital dialect expressions are best captured by two interrelated experiential dimensions: “emotional contagion and expressive intensity” and “social identity and interaction propensity.” A significant positive correlation was found between these two dimensions. suggesting a strong association between emotional experience and perceived social connectedness in digital dialect interactions. In addition, dialect familiarity and usage frequency exhibited weak correlations with certain experiential dimensions, while gender differences were not statistically significant. This study demonstrates that dialectal expressions on digital platforms are no longer merely markers of regional identity; they have become digital expressive resources that carry both emotion-transmission and relationship-building functions. These findings provide new empirical evidence for understanding the relationships among dialect communication, emotional interaction, and social identity in digital media environments.

## 1 Introduction

The proliferation of short-video platforms, real-time social media, and algorithm-driven recommendation systems have catalyzed significant transformations in digital language practices (Li and Shen, 2025). In contrast to traditional written language, which prioritizes standardized expression in communicative transmission, language use on social media increasingly exhibits features of orality (Lee and Wei, 2025), emotionality (Xue et al., 2025), and stylization (Doreleijers and Swanenberg, 2023). Users not only convey information through linguistic content but also employ linguistic forms to express emotions, establish interactional relationships, and shape social identities (Wei and Hua, 2019). Within this context, dialects—constituting linguistically resources imbued with regional cultural attributes and oral interactional characteristics—have progressively migrated from conventional spoken contexts into digital writing spaces, giving rise to platform-specific expressive practices in social media environments (Hatem et al., 2025).

In recent years, the phenomenon of digital dialect writing has continued to grow on Chinese social media platforms (Tian and Hao, 2025). Particularly in short-video comments, danmaku interactions, online chats, and entertainment-driven expressions, users frequently engage in dialectal expression through transliteration, homophonic substitution, dialectal lexicons, intensification of affect, symbolic mixing, and code-switching (Zhang and Ren, 2024). This trend not only reflects the written transformation of dialects in digital media but also indicates a shift from conventionally standardized writing toward emotionally expressive and interaction-oriented modes of communication. Compared to standard languages such as Mandarin, dialectal expressions typically carry stronger oral rhythms, contextual familiarity, and regional cultural color, thereby more readily fostering a sense of authenticity, co-presence, and interactional intimacy within social media environments (Wu and Liang, 2025).

Existing research has extensively addressed online dialect phenomena from perspectives spanning sociolinguistics, digital communication, and media culture (Sandow et al., 2026). The relevant literature generally recognizes that dialect writing in digital contexts constitutes not only a reproduction of regional language but also a crucial means through which users perform identity, engage in ingroup interaction, and enact cultural expression. One strand of research focuses on the cultural identity function of dialects in cyberspace, arguing that dialects reinforce place attachment and group boundaries (Li and Shen, 2025); another strand emphasizes the emotional transmission properties of dialects, pointing to associations between non-standard linguistic forms and emotional contagion as well as social engagement in interaction (Wu and Liang, 2025). Furthermore, studies on linguistic stylization, audience design, and platform interaction logic (Wang et al., 2023) further demonstrate that linguistic forms themselves have become significant social resources in digital interaction.

Nevertheless, three shortcomings persist in the existing literature. First, most current research on digital dialects remains centered on the classification of linguistic forms, regional cultural analysis, or social semiotic interpretation (Lee, 2025; Zhang and Ren, 2024), with relatively limited attention to how dialectal expressions are experientially perceived and evaluated by users. Second, although some studies touch upon issues of emotional expression, social identity, or interactional behavior, the relevant analyses often remain at the level of theoretical speculation or textual interpretation (Li and Shen, 2025; Wei and Hua, 2019), lacking empirical testing based on user perception data. Third, the extant literature typically treats emotion (Goldenberg and Gross, 2020), identity (Przymus et al., 2022), and interactional propensity (Imuta and Spence, 2020) as relatively independent conceptual dimensions, seldom examining the interwoven relationships and overall structure of these experiential dimensions in digital interaction.

The linguistic ecology of digital dialect interaction in the Xinjiang context presents a more intricate scenario. The linguistic ecology perspective posits that language forms are not isolated entities but dynamic systems continuously shaped and evolving within specific social environments, cultural relationships, and processes of language contact (Bastardas-Boada, 2019). As a pivotal region in western China characterized by extensive multi-ethnic and multilingual contact, Xinjiang's linguistic landscape has been persistently influenced by inter-ethnic communication, the amalgamation of regional cultures, and cross-regional population mobility (Qian, 2022). Within this linguistic ecological framework, Xinjiang Chinese dialects exhibit not only lexical, phonetic, and expressive variations across locales but also encapsulate local lived experiences, group dynamics, and cultural significance. In contrast to Putonghua, which is more strongly associated with cross-regional communication and standardization, Xinjiang Chinese dialects are more deeply embedded with local cultural experiences and emotional communication modalities in everyday interactions. Consequently, they may manifest distinct social meanings and psychological perceptions within digital environments.

The selection of Xinjiang Chinese dialects as the research subject is motivated not only by their regional specificity but also by their complex language contact background, which offers a typical research scenario for examining how linguistic variants carry social meaning, emotional expression, and interactional relationships. Compared to dialect usage in a monolingual environment, Xinjiang digital dialect practices can illustrate how linguistic variants are reinterpreted and disseminated within a multicultural confluence. Furthermore, the algorithmic recommendation mechanisms, entertainment-driven dissemination logic, and high-frequency interaction structures characteristic of social media platforms (Chen et al., 2024) have amplified the visibility of local linguistic expressions, facilitating their transformation from offline communicative resources into digital interactive ones. Therefore, Xinjiang digital dialect practices represent not only a shift in linguistic forms but also reflect the interrelationship between linguistic ecology, digital media environments, and socio-psychological experiences. Examining the emotional expression, social connection, and interactional tendencies of Xinjiang Chinese dialects in digital interactions from this perspective will contribute to a deeper understanding of how linguistic variants convey affective meaning, local identity, and social relationships in contemporary communication environments.

Against this background, the present study takes Xinjiang Chinese dialect expressions in social media as its object of investigation (Figure 1), combining qualitative textual analysis with quantitative perceptual assessment to explore the core experiential structures underlying digital dialect interaction. The study first conducts a thematic analysis of social media corpora to identify key linguistic practices in digital dialect writing; it then employs experimental questionnaires and exploratory statistical analysis to examine participants' perceptual patterns of dialect expressions and their underlying experiential dimensions. Unlike previous research that treats dialect primarily as a marker of regional identity (Imuta and Spence, 2020; McKenzie, 2019), this study focuses on the experiential perceptual structure of dialect expressions in digital interactive contexts, with particular attention to the roles of dialect in emotional contagion, interactional proximity, and social identification. The results indicate that participants' perceptions of digital dialect expressions are best understood as comprising two interrelated experiential dimensions: “emotional contagion and expression intensity” and “social identity and interactional tendency.” These findings contribute to a deeper understanding of dialect diffusion mechanisms on digital platforms and provide new empirical evidence concerning the relationships between emotional interaction, social connection, and non-standard language practices in digital language research.

**Figure 1:**
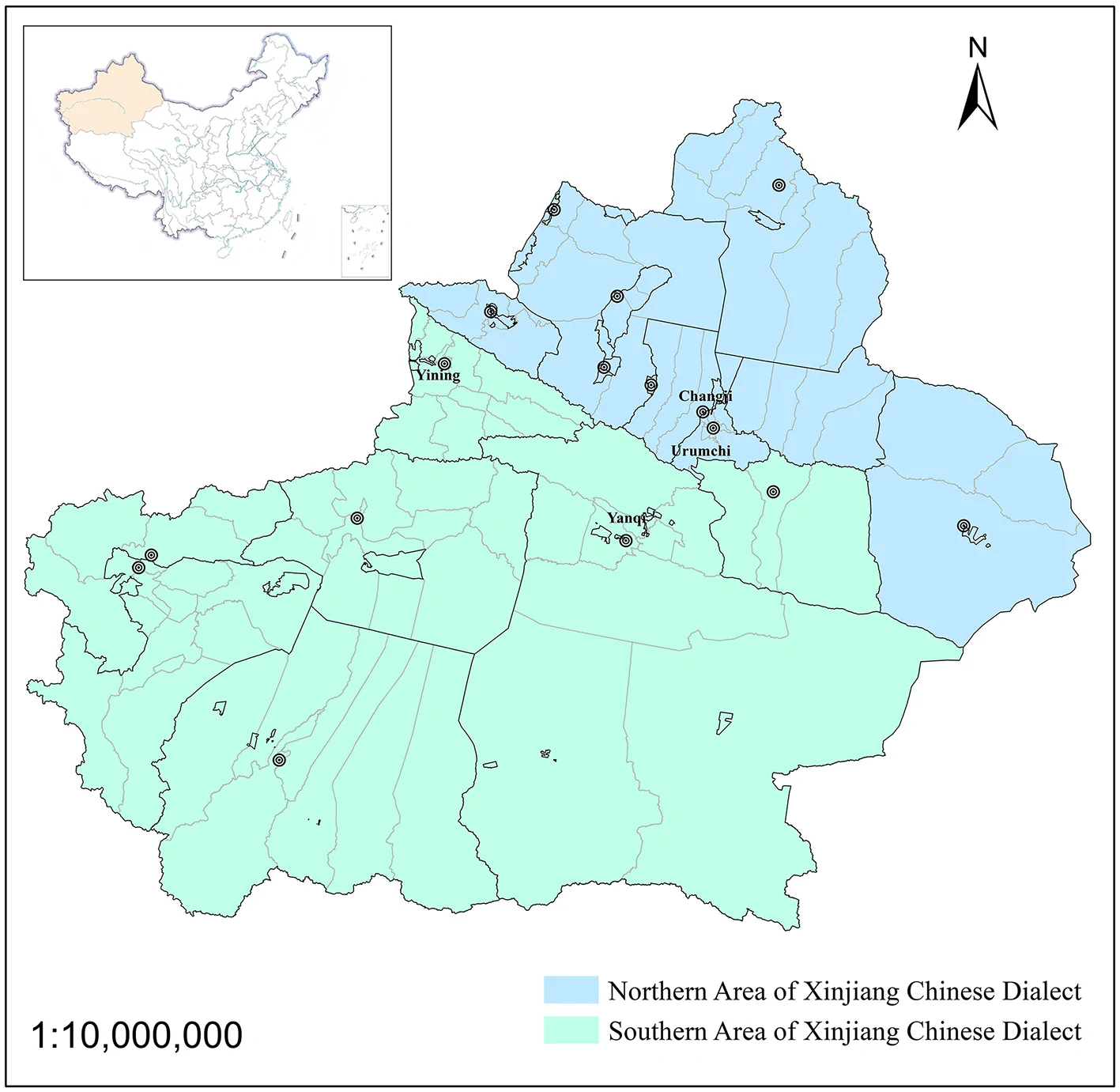
Map of Xinjiang Chinese dialect locations. Location of the study area in Xinjiang, China. The map was independently redrawn and simplified by the authors based on publicly available standard map information and is intended solely to illustrate the geographical location of the study area.

## 2 Literature review and theoretical framework

### 2.1 Digital dialect interaction in new media communication contexts

With the proliferation of social media, short-video platforms, and instant interactive media, dialects have progressively transcended traditional oral communication domains, entering the spheres of digital writing and online interaction. Within new media contexts, language not only fulfills the function of information transmission but has also increasingly become a critical medium for emotional expression (Rahmani et al., 2019), interaction construction, and social relationship maintenance (Zhang and Zhang, 2026). Compared to standardized linguistic forms, dialectal expressions typically exhibit stronger oral rhythms, contextual familiarity, and expressive tension, thereby facilitating emotional contagion and interactional closeness in digital interactions.

From the perspective of language variation theory, linguistic differences manifest not merely as formal changes at the phonetic, lexical, or grammatical levels, but are intrinsically linked to specific social contexts, group dynamics, and communicative objectives (Hazen, 2017). Distinct linguistic varieties frequently carry particular social meanings and are interpreted in terms of identity, affect, and relational aspects during actual communication. Furthermore, language indexicality theory posits that linguistic forms not only convey literal meaning but also point to broader social information, such as geographical origin, group affiliation, cultural experiences, and interactional relationships (Ferrara and Hodge, 2018). In this regard, dialects, as linguistically varied forms embedded in specific locales, derive their social functions not solely from their inherent linguistic structures but from a shared system of meanings cultivated through prolonged social usage. Unlike standardized languages such as Mandarin, dialects are more closely tied to local lived experiences, collective cultural memories, and habitual group interactions, thus more readily evoking users' feelings of familiarity, closeness, and emotional attachment.

Prior research has indicated that non-standard forms of expression in online language—such as colloquial spellings, hybrid symbols, transliterated expressions, and stylized language—are associated with emotional perception and participatory experience in interactions (Albawardi, 2018). Such expressions not only help reduce interactional distance but also strengthen the communicative atmosphere and emotional connections among users. In social media environments, linguistic form itself has gradually become part of the interactional experience, with its function extending beyond semantic transmission to serve as an emotional and relational communicative resource. Concurrently, digital language practices possess prominent social identity attributes (Zhang and Zhang, 2026). Social identity theory posits that individuals tend to reinforce in-group identification through shared symbols, common modes of expression, and group-based linguistic resources (Abrams and Hogg, 1987). In online interactions, specific linguistic styles enable users to rapidly identify group boundaries, enhancing familiarity and interactional proximity (Hu, 2024). As a linguistic resource imbued with local cultural color and shared experiential backgrounds, dialects more readily foster an in-group perception of communication in digital interactions.

Although existing research has extensively examined the relationships among online language (Li and Shen, 2025), emotional contagion (Goldenberg and Gross, 2020), and identity (Davis et al., 2019), studies on digital dialect interaction remain largely confined to sociolinguistic or descriptive analyses of linguistic forms (Wei, 2020). Systematic empirical research on how dialectal expressions influence emotional experience and social identity perception in digital interactions remains lacking. In particular, empirical testing of the experiential structure of digital dialect interaction under controlled experimental conditions is still relatively limited (Walton, 2022). Accordingly, this study takes the digital interaction practices of Xinjiang Chinese dialects in new media contexts as its research object, focusing on two core experiential dimensions of dialectal expression in digital interactions: first, “emotional contagion and expression intensity,” and second, “social identity and interactional tendency.” It attempts to conduct an exploratory analysis of the experiential structure of digital dialect interaction through a combination of qualitative analysis and experimental data.

### 2.2 Emotional contagion and expressive intensity: affective experiences in digital language interaction

Affective theory posits that linguistic forms not only shape information comprehension but also influence individuals' emotional arousal, emotional intensity, and interactive experiences (Hatfield et al., 1993; Russell, 2003). Within digital communication environments, the tonal style, rhythmic pacing, and affective coloring of language can directly affect users' emotional perception and interactional responses (Lambton-Howard et al., 2020). Compared to standardized expressions, dialects typically exhibit more pronounced oral features and a colloquial character, rendering them more readily perceived as “authentic”, “lively”, or “emotionally warm” modes of expression (Al-Harahsheh et al., 2025; Martin and Jenkins, 2024). In social media contexts, such expressive characteristics are associated with heightened evaluations of emotional contagion, thereby enhancing users' emotional resonance and interactive engagement. Prior research has indicated that emotional transmission in online interactions is not solely dependent on semantic content but is also closely linked to linguistic style, expressive form, and contextual familiarity (Pfeiffer et al., 2023). Particularly in real-time commentary, short-video interactions, and lightweight social expressions, users tend to exhibit stronger emotional responses to expressions marked by local oral features. Consequently, emotional experiences in digital dialect interactions are manifested not merely as semantic understanding but also as a situational, interactive perceptual process. On the basis of the foregoing research, this study conceptualizes “emotional contagion and expressive intensity” as a critical experiential dimension in digital dialect interactions, focusing primarily on the experiential characteristics of dialectal expression with respect to emotional arousal, perceived vividness, and interactive appeal.

### 2.3 Social identity and interactional tendency: relational experiences in digital dialect interactions

Beyond emotional experiences, digital dialect interactions also entail pronounced features of social identity and relational interaction (Davis et al., 2019). Social identity theory posits that individuals strengthen their sense of group belonging through shared symbols and group-based modes of expression, and in the process of interaction, they form distinctions between in-groups and out-groups (Imuta and Spence, 2020; Tajfel and Turner, 2004). In digital social contexts, dialect functions not only as a regional linguistic resource but also as an interactional symbol with social identification capabilities (Elleström, 2022). Through dialectal expression, users can rapidly establish familiarity and approachability in interactions, thereby fostering a shared communicative atmosphere within specific circles. Particularly in social interactions imbued with regional cultural backgrounds, dialectal expression often reinforces experiential perceptions of “localness,” “insiderness,” or “in-group communication” (Champoux-Larsson and Zakrisson, 2026). Existing studies have demonstrated that linguistic choices on digital platforms not only reflect communicative content but also encode users' interactional positioning and social relationship construction (Burgers and Beukeboom, 2020). To a certain extent, dialectal expression enhances willingness to engage interactively, increases communicative approachability among users, and facilitates relational bonding in social interactions. Therefore, this study further identifies “social identity and interactional tendency” as another core experiential dimension in digital dialect interactions, with a focus on the experiential characteristics of dialectal expression in terms of group familiarity, willingness to participate interactively, and social approachability.

### 2.4 Theoretical framework and research approach

Building on the aforementioned studies, this research conceptualizes digital dialect interaction as a communicative practice jointly shaped by emotional experience (Goldenberg and Gross, 2020) and social identity (Przymus et al., 2022). In contrast to prior work that treats dialect as a static marker of geographic origin (McKenzie, 2019), this study foregrounds the experiential role of dialect within digital interaction—specifically, how dialect influences users' emotional experience, interactional perception, and social connectedness in new media communication environments. We contend that the new media communication context provides specific situational conditions for digital dialect interaction, including real-time interaction, emotionally charged communication, and platform-mediated dissemination structures. Against this backdrop, dialectal expression does not merely manifest as formal linguistic variation; it may further shape users' overall experiential perception of emotional contagion, vividness, local familiarity, and interactional willingness. Integrating preliminary qualitative findings with subsequent experimental analyses, this study distills the experiential structure of digital dialect interaction into two interrelated core dimensions: “emotional contagion and expressive intensity” and “social identity and interactional tendency.” The former corresponds primarily to the emotional experience pathway in digital interaction, while the latter corresponds to the relational experience pathway. Together, these dimensions constitute the core experiential structure of digital dialect interaction and provide a theoretical foundation for subsequent empirical analysis (Figure 2). Simultaneously, this study situates the aforementioned digital interaction processes within the language ecology of the Xinjiang region. Against the backdrop of the coexisting promotion of Standard Chinese and the continued use of local dialects, Dialectal expressions on digital platforms reflect not only changes in linguistic form but also a reorganization of emotional experience and social connectedness in digital interaction.

**Figure 2:**
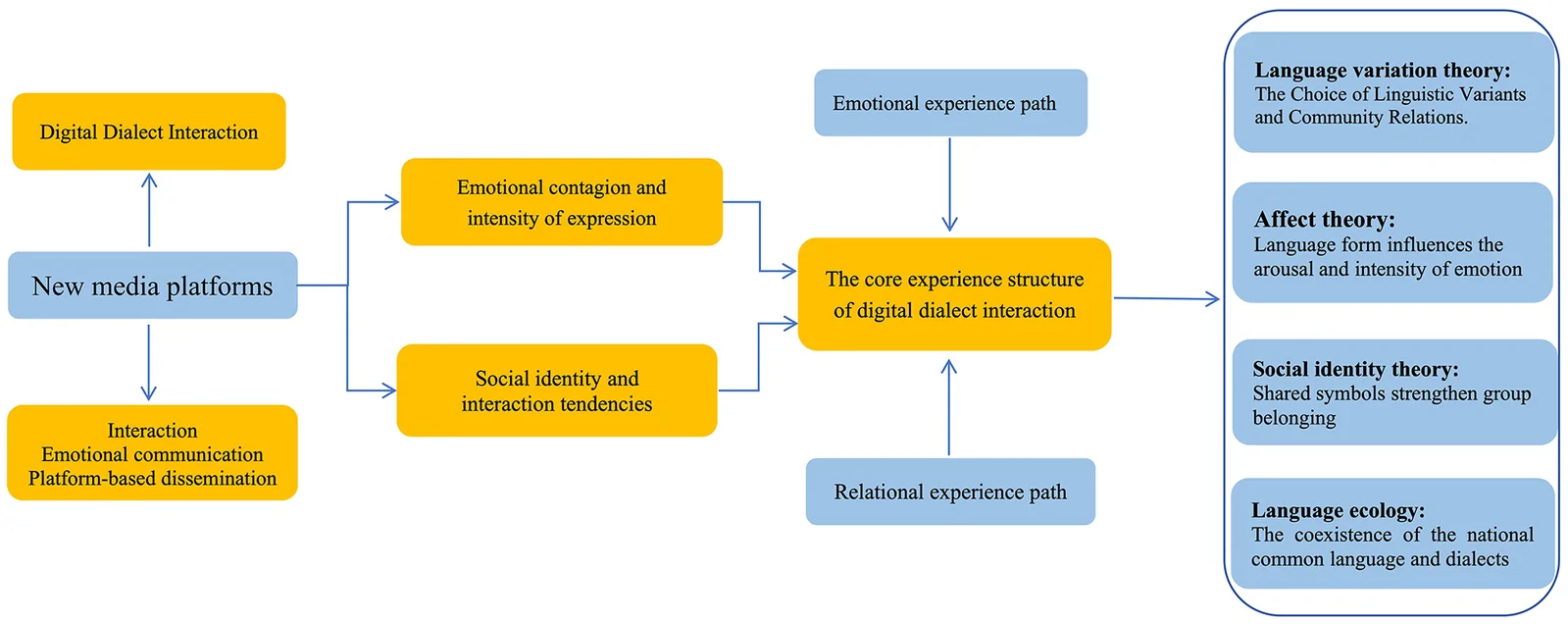
Research framework model. The framework was developed by the authors based on relevant theoretical perspectives and the empirical focus of this study, illustrating how digital dialect practices relate to emotional experience, social connectedness, and interactional tendencies.

## 3 Methods

### 3.1 Research design

This study employed an exploratory sequential mixed-methods design (Demkowicz et al., 2025; Wess et al., 2022), following a two-phase “qualitative analysis–perception experiment” approach to comprehensively investigate the written use of the Xinjiang Chinese dialect in social media contexts and its associated psychological perceptions. The overarching objective was not to test strictly causal relationships, but rather to examine patterns of association between different linguistic forms and emotional experience, social connectedness, and interaction tendencies. The study adopted a “qualitative-led quantitative follow-up” path. In Study 1, open coding, axial coding, and thematic synthesis (Scott, 2004) of dialectal interaction texts from Xinjiang social media yielded 53 initial coding categories and six core themes. These themes primarily pertained to emotional expression, interaction atmosphere, group belonging, local familiarity, and social interaction tendencies.

Rather than being directly translated into measurement items or used solely to establish the scale structure, the qualitative findings primarily served to identify core linguistic practices, interactional contexts, and experiential dimensions in digital dialect use—offering contextualized references for selecting stimuli, defining constructs, and designing measures in Study 2. The measurement instruments in Study 2 were primarily developed by integrating relevant theoretical constructs from existing research on digital language, emotional interaction, and social relationships, and were adapted contextually based on the linguistic practices illuminated by the qualitative findings. Consequently, this study adopted a “theory-driven quantitative design inspired by qualitative discoveries,” rather than a straightforward path of “transforming qualitative coding results into a scale.”

In the first phase, qualitative analysis identified typical expressive features, interactional contexts, and linguistic practices of the Xinjiang Chinese dialect on social media. On this basis, thematic cues related to emotional expression, regional perception, social connectedness, and interaction tendencies were extracted. These results were then used in the quantitative phase to screen stimulus materials, define measurement dimensions, and generate item content, thereby forming a bridging pathway from “qualitative findings to quantitative measurement”.

In the second phase, a repeated-measures perception experiment was employed to compare participants' subjective perceptions of different linguistic forms (Xinjiang Chinese dialect vs. Mandarin Chinese). The study focused on four psychological dimensions: emotional intensity (Russell, 2003), perceived locality (Imuta and Spence, 2020), social connectedness (Davis et al., 2019), and interaction intention (Wu and Liang, 2025). The quantitative phase served primarily to further examine and supplement the linguistic features and interactional trends identified in the qualitative phase.

To minimize the influence of semantic content differences on the results, the study constructed paired stimulus materials (Wu et al., 2024) comprising “dialect version of the same semantic content-Mandarin Chinese version,” thereby making linguistic form the primary comparative dimension. The overall study aimed to explore, from the perspective of linguistic practice and psychosocial correlates, the emotional and interactive characteristics of dialect expressions in digital contexts.

### 3.2 Study 1: qualitative analysis of dialect writing on social media

#### 3.2.1 Data sources and sample composition

This study focuses on the written practices of Xinjiang Chinese dialects on mainstream social media platforms in China. To enhance the diversity and ecological validity of the corpus, this research selected seven representative types of social media platforms, including short-video platforms, video communities, social interaction platforms, lifestyle service platforms, and news discussion platforms, and analyzed their public interactive texts (Table 1). Platform selection was primarily based on three criteria: (1) platforms with high user engagement, capable of generating rich user-generated content; (2) platforms with strong interactive attributes, where users can engage in linguistic communication through comments, replies, bullet comments, and other means; (3) differences in content production mechanisms and interaction models across platforms, which facilitate observation of Xinjiang Mandarin dialect expression in different digital communication scenarios.

**Table 1:** Research data sources.

Platform	Category	Data type	User base (100 million)	Percentage
Douyin	Video	Comments, program titles, program descriptions, etc.	7.72	35%
Bilibili	Video	Comments, bullet comments, program titles, program descriptions, etc.	1.02	10%
Meituan	Lifestyle services	Comments, titles, descriptions, etc.	4.61	15%
Dianping	Lifestyle services	Comments, titles, descriptions, etc.	1.11	5%
WeChat	Social	Comments, titles, descriptions, etc.	10.53	25%
Weibo	Social	Comments, titles, descriptions, etc.	4.81	8%
Toutiao	News	Comments, program titles, program descriptions, etc.	3.77	2%

Short-video and video community platforms showcase how dialects are used in entertainment dissemination, comment interaction, and emotional expression; social interaction platforms better reflect dialect writing practices in daily communication; lifestyle service and news discussion platforms provide samples of local language expression in diverse social contexts. Consequently, the multi-platform corpus design avoids the limitations of a single platform ecosystem on research findings and more comprehensively presents the diversified practices of dialect expression in digital environments.

Given that content presentation on digital platforms is influenced by recommendation algorithms, interaction popularity, and platform visibility mechanisms, the corpus acquired in this study reflects users' linguistic practices in digital interactions and may also be affected by platform algorithmic amplification effects. Therefore, digital dialect expressions in this research are not entirely equivalent to all linguistic usage in a natural state but are closer to visibility-based linguistic practices within a platform-mediated communication environment.

The corpus was collected between January 2020 and September 2024. The study employed a time-interval sampling method, selecting four random days per week for data collection, during which trending videos and their comment sections from the platform's homepage recommendations were observed. On each sampling day, the first six updated videos were selected, while videos with a duration of fewer than 15 s were excluded to ensure the integrity of the interactional context.

The study focused on user-generated content exhibiting prominent features of the Xinjiang Chinese dialect. The initial inclusion criteria were: (1) the presence of identifiable dialectal vocabulary, phonological transcriptions, or region-specific expressions; (2) a complete interactional context; and (3) the text demonstrated actual social communicative functions. The following exclusion criteria were also established: (1) texts with insufficient semantic content or those consisting solely of emojis; (2) platform auto-replies or commercial promotional content; and (3) general internet catchphrases lacking notable regional linguistic features.

The final corpus comprised 965 valid textual instances. It should be noted that during the subsequent open coding phase, five texts were excluded from the final coding analysis due to repetitive expressions and overlapping information; thus, the actual unit of analysis for the open coding phase was 960 linguistic instances. Throughout the study, the unit of analysis was the “textual instance” (Figure 3).

**Figure 3:**
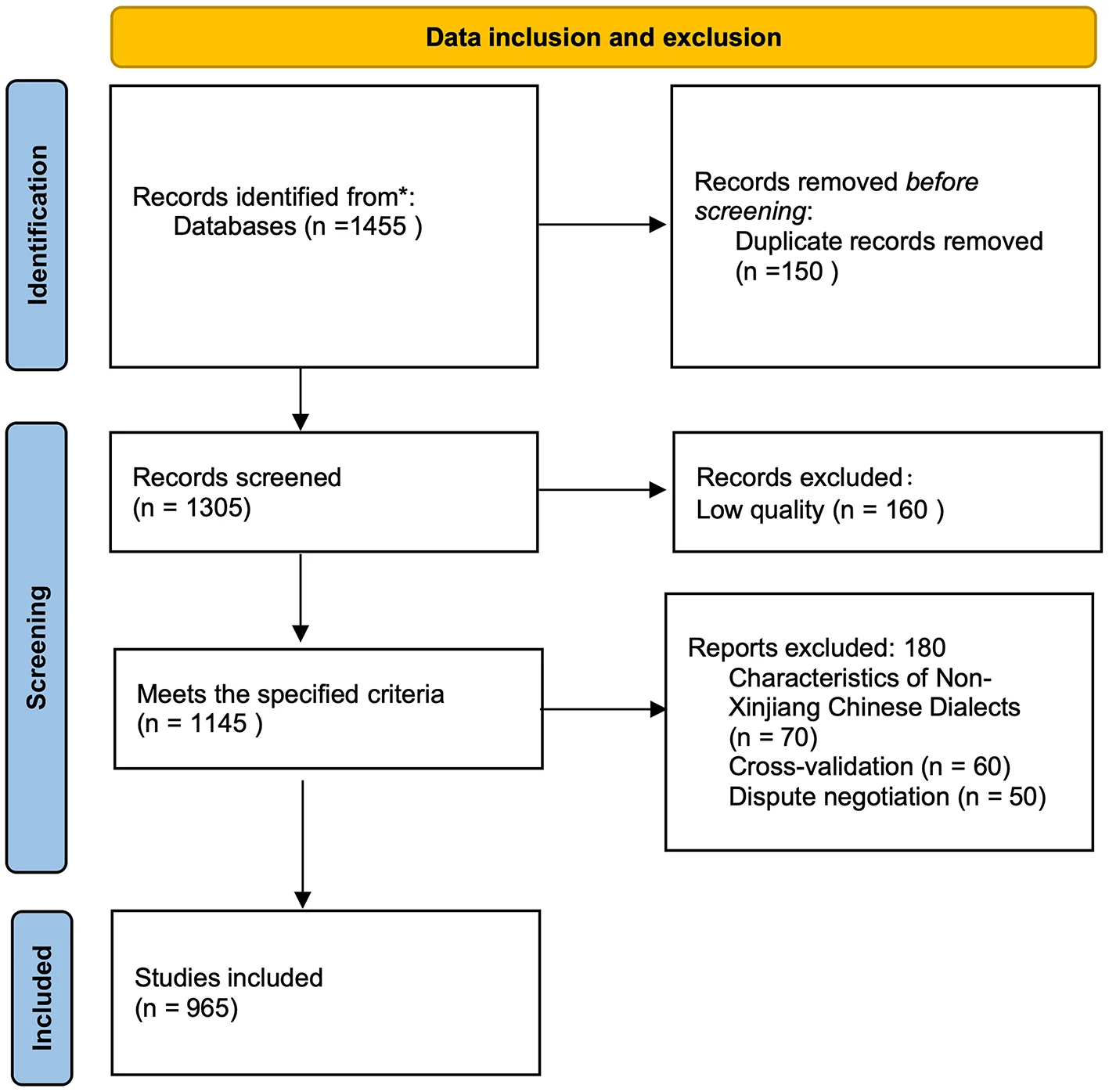
Data screening flowchart.

Additionally, because the research corpus was primarily derived from platform-recommended and trending interactive content, the data may have been influenced by the platform's algorithmic recommendation mechanisms. Consequently, the linguistic practices documented in this study reflect both users' natural expressions and, to some extent, the amplification effects of the platform's algorithm on highly interactive content.

#### 3.2.2 Data processing and coding procedures

The qualitative analysis in this study was primarily conducted using a reflexive thematic analysis approach (Braun and Clarke, 2019). Coding was carried out by the first author, with ongoing discussions within the research team throughout the analytical process to dynamically revise and refine the coding framework and thematic structure. The research team engaged in multiple rounds of discussion focusing on coding boundaries, thematic classification, and conceptual interpretation to enhance the transparency and interpretive consistency of the analysis. As this study did not employ a strict independent double-coding procedure, formal inter-coder reliability statistics (e.g., Cohen's κ or Krippendorff's α) were not computed. Instead, the study emphasizes interpretive consistency, constant comparison, and a reflexive analytical process grounded in team collaboration, rather than treating statistical agreement as the sole quality criterion.

The coding process comprised three stages: open coding, axial coding, and selective coding (Scott, 2004). First, during open coding, the research team conducted a line-by-line analysis of the linguistic forms, pragmatic strategies, and interactional patterns present in the corpus, assigning initial conceptual labels. Second, in the axial coding stage, concepts with similar characteristics were aggregated and categorized to further refine the core categories of digital dialect interaction. Finally, in the selective coding stage, the relationships among different categories were integrated, gradually constructing an overarching interpretive framework of “dialectal features—interactional strategies—social media expression practices”.

The coding focus of this study centered on the linguistic practices of digital dialects, including phenomena such as dialectal lexicon, transliterated forms, prosodic expressions, symbol blending, code-switching, and interactional style. Accordingly, the resulting coding outcomes primarily reflect the specific usage patterns and interactional features of digital dialects on social media. Discussions pertaining to emotional expression, social connectivity, and interactional tendencies in subsequent sections are interpretive analyses based on the language use context and subsequent quantitative findings, rather than psychometric measures derived directly from the qualitative coding process. To enhance research credibility, the research team conducted multiple rounds of discussion and revision during the open coding, axial coding, and thematic abstraction stages, and repeatedly cross-checked core themes against representative corpus examples. Additionally, a comprehensive coding log, thematic abstraction process, and mapping between the corpus and codes were maintained throughout the research to strengthen the traceability and transparency of the analytical process. The research team has had prolonged exposure to the linguistic and cultural environment of Xinjiang and possesses recognized expertise in identifying relevant dialectal expressions and social media contexts. As coding progressed, once newly added texts no longer yielded distinctly new thematic categories, the study was considered to have reached a sufficient state of thematic saturation.

### 3.3 Study 2: quantitative perceptual study of dialect expressions

#### 3.3.1 Study design and participants

The study employed a repeated-measures design (Jordan et al., 2026). All participants completed evaluation tasks for all stimulus materials. Data from a total of 149 participants were included in the final analysis. Participants were primarily recruited through university campus networks, student social channels, and public calls by the research team, utilizing a convenience sampling method (Stratton, 2021). The sample pre-dominantly comprised young individuals from universities, with ages concentrated between 18–21 years, and primarily consisted of participants at the college/undergraduate level. The selection of this demographic was based on the following considerations: firstly, young individuals are frequent users of social media and digital communication platforms, possessing extensive experience with online language; secondly, this group is more likely to encounter and engage in scenarios involving short video comments, instant messaging, and other forms of digital dialect interaction, making them suitable as subjects for exploring perceptual experiences of digital dialects.

All participants met the following inclusion criteria: (1) daily social media usage experience; (2) ability to comprehend Mandarin Chinese expressions; (3) willingness to complete all experimental tasks. While some participants possessed varying degrees of familiarity with Xinjiang Chinese dialects, no participants were required to have a Xinjiang dialect background. During the experiment, each participant was required to evaluate stimulus materials in both dialectal and Mandarin versions. All stimulus materials were presented in a paired design, with each set comprising a Xinjiang Mandarin dialect version and a semantically equivalent Mandarin version. The experiment included 10 sets of stimulus pairs. The order of stimulus presentation was randomized to mitigate the impact of order effects; concurrently, dialectal and Mandarin versions were presented non-contiguously to minimize explicit judgment bias that might arise from direct comparison.

#### 3.3.2 Stimulus materials and scale development

The experimental stimulus materials were derived primarily from the high-frequency dialect expressions and typical social media interaction contexts identified in Study 1. The research team selected representative expressions from the qualitative corpus and constructed corresponding Mandarin versions while striving to maintain semantic consistency. However, owing to the distinct regional cultural characteristics of certain Xinjiang Chinese dialect vocabulary, achieving full equivalence in emotional coloring and interactional tone between the dialect and its Mandarin counterpart proved challenging (Wu et al., 2024). Accordingly, “semantic consistency” in this study refers to consistency in core semantic content rather than complete linguistic equivalence.

The measurement instrument employed in this study is an exploratory tool developed by the research team specifically for the context of digital dialect interaction. Its objective is to evaluate participants' subjective perceptions of diverse linguistic expressions. The development of this scale draws comprehensively from existing theoretical research on emotional expression, social identity, social connection, and online interaction. Concurrently, it integrates the distinctive features of digital dialect practices identified in Study One, with contextualized adjustments made to the measurement content. Consequently, the scale's items primarily originate from three sources: (1) relevant studies on emotional expression, social identity, and online interaction; (2) typical linguistic practices and interaction themes presented in Study One; and (3) actual expressive contexts of Xinjiang Chinese dialects within social media.

During item development, the team first constructed an initial item pool organized around four dimensions: emotional expression, local perception, social connectedness, and interaction tendency. Subsequently, three scholars with expertise in sociolinguistics, psychology, and mixed-methods research were invited to review the items (Conry-Murray et al., 2024), focusing on clarity of phrasing, conceptual relevance, and contextual appropriateness. Based on their feedback, the wording of certain items was revised, and redundant expressions were eliminated.

Prior to the formal experiment, a small-scale pilot test was conducted with a group of university students who had experience using social media. Based on their feedback, items with ambiguous phrasing were linguistically adjusted to enhance comprehensibility and reading fluency. The final scale comprises 9 Likert-type items (Emerson, 2017), utilizing a 5-point response format (1 = Strongly Disagree, 5 = Strongly Agree). Table 2 presents definitions and scopes for a selection of representative items; all items are provided in Supplementary material 1.

**Table 2:** Overview of questionnaire constructs and representative items.

No.	Dimension	Definition	Representative statements
1	Emotional intensity	The perceived emotional strength and vividness elicited by the language expression.	This expression feels emotionally vivid; This expression conveys stronger emotional feelings.
2	Perceived localness	The perceived degree of regional and local characteristics conveyed by the expression.	This expression reflects local linguistic features.
3	Social connectedness	The perceived familiarity and social closeness associated with the expression	This expression sounds more like something “one of us” would say.
4	Interaction intention	The willingness to engage in further communication.	I would like to respond or interact with this expression on social media.

#### 3.3.3 Experimental procedure and stimulus presentation

All 149 participants completed the rating task for all 10 stimulus pairs. A total of 20 experimental stimuli were involved. Each stimulus set comprised a Xinjiang Mandarin dialect version and its corresponding Standard Mandarin version. To mitigate explicit judgment bias arising from direct comparison, the dialect and Standard Mandarin versions were presented as independent stimuli within the questionnaire, rather than side-by-side on the same page. All stimuli were randomized using the questionnaire system's built-in randomization function to minimize the impact of order effects and fatigue on the rating results. The questionnaire was distributed electronically via an online survey platform. Participants accessed the experiment through a questionnaire link on a webpage and completed the evaluation task anonymously. The survey system automatically recorded responses upon completion.

After reading each stimulus, participants rated all nine evaluation items. All items were measured on a 5-point Likert scale (1 = “strongly disagree”, 5 = “strongly agree”). The experimental stimuli were primarily drawn from authentic interactive expressions on Xinjiang social media platforms, including short video comments, instant messaging, and online colloquial speech. The research team first constructed an initial corpus, then screened candidate expressions based on the following criteria: prominent dialectal features, functional semantic correspondence achievable in Mandarin, interactive discourse properties, and avoidance of overly obscure expressions. Through this process, ten dialect–Mandarin stimulus pairs were ultimately formed.

#### 3.3.4 Variable measurement and structural validation

Based on prior theoretical analysis and research design, this study focuses on examining four psychological perception dimensions: (1) emotional intensity, defined as the subjective degree of emotional arousal experienced when encountering a linguistic expression; (2) perceived locality, referring to the subjective perception of the expression's regional characteristics and local flavor; (3) social connectedness, encompassing the sense of familiarity, closeness, and group proximity elicited by the expression; (4) interaction willingness, operationalized as the individual's subjective tendency to further comment, reply, or engage interactively. To validate the scale structure, an exploratory factor analysis (EFA) was conducted on the nine items (Rogers, 2024). Prior to the analysis, the suitability of the data for factor analysis was assessed using the Kaiser–Meyer–Olkin (KMO) test and Bartlett's test of sphericity. Factor extraction was performed using the principal axis factoring method with oblique rotation, thereby allowing correlations among the psychological dimensions. The factor loadings, cross-loadings, and internal consistency indices for each item are reported to evaluate the adequacy and stability of the scale structure. Given that the present scale was developed specifically for the Xinjiang Chinese dialect social media context, and that no fully corresponding validated Chinese measurement instruments currently exist, a formal convergent validity test was not pursued. Future research should further validate the scale using broader instruments for online emotional expression or social connectedness.

#### 3.3.5 Experimental materials and paired design

This study employed a within-subject paired design to compare psychological perception differences between Xinjiang Chinese dialect expressions and Standard Mandarin expressions in digital social contexts. The experimental materials consisted of 10 sets of social media linguistic stimuli, each set containing one Xinjiang Chinese dialect version and its corresponding Standard Mandarin version, yielding a total of 20 experimental stimuli. All participants were required to read and evaluate all stimulus items. To mitigate order effects, the presentation sequence of stimuli was randomized across the questionnaire.

Each stimulus set was designed to maintain comparable core semantic content, differing only in linguistic form—either Xinjiang Chinese dialect or Standard Mandarin. For example, in certain stimuli, standard expressions in Mandarin were replaced with colloquial expressions, modal particles, or region-specific terms characteristic of the Xinjiang Chinese dialect, while preserving the original communicative intent. It should be noted that, due to the strong local cultural connotations and context dependency of some Xinjiang Chinese dialect expressions, their Standard Mandarin counterparts can only achieve functional semantic equivalence (Wu et al., 2024), rather than strict linguistic equivalence in terms of word-for-word translation. Accordingly, the paired stimuli in this study emphasized approximate consistency in communicative function and core semantics, rather than exact lexical correspondence.

After reading each stimulus, participants rated their subjective perceptions on a Likert scale. The initial item set focused on experiential features such as emotional contagion, interaction tendency, local familiarity, and group identification. Subsequent exploratory factor analysis revealed that these items coalesced into two interrelated core experiential dimensions: “Emotional Contagion and Expressive Intensity” and “Social Identification and Interaction Tendency.”

### 3.4 Data analysis

All data were organized and analyzed using SPSS 27.0 (IBM Corp., Armonk, NY, USA). First, descriptive statistics—including means, standard deviations, and 95% confidence intervals—were computed for each variable to illustrate general distribution characteristics under different linguistic conditions. Second, for the repeated-measures design, a repeated-measures analysis of variance (Ziegel and Girden, 1993) was conducted with language form (dialect/Mandarin) and stimulus pair as within-subject factors to examine perception differences across expression conditions. Accordingly, analyses were performed separately for the identified psychological dimensions based on the scale structure. In addition, exploratory analyses were carried out for demographic variables, including gender, education level, dialect familiarity, and frequency of use. Given the small sample sizes for some subgroups, the relevant results are reported primarily as supplementary observations, without strong causal interpretation. To enhance research transparency, the anonymized data and analysis scripts have been uploaded to the Open Science Framework (OSF) platform (https://osf.io/df3m6?view_only=1f14b9f30d6d4454b3d541f8e7e1a0a4). All statistical results were rechecked to ensure consistency between descriptive and inferential statistics.

### 3.5 Ethics statement

This study has been reviewed and approved by the Ethics Committee of Xinjiang University (Approval No. XJSF2026A51). All participants read the study information and signed informed consent forms prior to participation. No personally identifiable information was collected during the study; all data were stored and analyzed in an anonymized form for academic research purposes only. The entire research procedure adhered to the ethical principles outlined in the Declaration of Helsinki to safeguard participants' right to informed consent and data security.

## 4 Results

### 4.1 Coding analysis of qualitative research

#### 4.1.1 Open coding

Open coding is the initial type of coding performed in a research project and involves unrestricted coding of data (Conry-Murray et al., 2024). This process compares events, actions, or interactions with similar ones to identify similarities and differences, groups analogous concepts under conceptual labels, and ultimately generates categories and subcategories. In this study, 960 instances of dialectal expressions were coded, and each instance was analyzed for its pragmatic features and related phenomena. Through iterative examination and analysis of each instance, 53 categories and initial concepts were abstracted from the textual data (Table 3). To conserve space, each initial concept is illustrated with a limited selection of original utterances.

**Table 3:** Open coding.

No.	Initial categories and frequencies	Partial raw data statements (initial concepts)
a1	Locally popular character forms (37)	Tai lao dao le [Lao dao: impressive]
a2	Homophone substitutions (20)	Tu da de hen [There's a lot of dirt.]
a3	Dialect-specific characters (33)	Ni ga jiu mu [Ga: small], Sao ni [Sao: impressive]
a4	Authentic characters from ancient texts (37)	Dai dai de [Very good]
a5	Simplified Chinese characters (254)	Song tai jia [Unpresentable]
a6	Traditional Chinese characters (10)	Po yi shi Qi tai zhi gu hua [Po yi: wife, old saying in Qitai County]
a7	Trademark names (2)	Picture format trademark: Mu ma da jiao ma ji [Mu ma da: no problem]
a8	Signboard names (3)	Picture format trademark: Lao dao niu ti [lao dao: impressive]
a9	Mixed use of Chinese characters and emoji (1)	Cong xiao yi ge bi wo tong tong zi li mian\”(·ε·‘^*^) gun dao da di [Rolling from the small quilt to the big one]
a10	Text emoji packs (9)	Kitten emoji featuring the text “po fan” [po fan: worry]
a11	Mixed use of Chinese characters and emoticons (47)	Wo kang bing zi! Jiu si zi yang kang di, huo mian di shi hou fang 4 ge ji de dan. [kang bing zi: grilled pancake]
a12	Combined use of numbers and Chinese characters (4)	San Shi Hei Li [It means the night of Chinese Lunar New Year's Eve.]
a13	Combined use of numerical homophones and Chinese characters (3)	ai 7 sa [7 sa: go]
a14	Combined use of Chinese characters and markers (8)	Ding…ding dun ha [Stay quiet.]
a15	Combined use of Chinese characters and dots (22)	xia ye zi gan![Work hard.]
a16	Combined use of Chinese characters and mathematical symbols (3)	Na=Ta (meaning: he/she)
a17	Chinese character transliteration (137)	Xing le sa [sa: ok]
a18	Pinyin transcription of dialect phonetic values (8)	Nia [He/she]
a19	Pinyin transcription of complex Chinese characters (19)	Na ke neng zen de bu xing [He might be very tired.]
a20	Combined use of pinyin transcription and homophonic Chinese characters (10)	Ni fo sa di ni[What are you talking about?]
a21	Romanized expressions for euphemisms (4)	zhe bi ping lun[bi: poor, inferior]
a22	Avoidance of homophonic archaic characters (2)	ba ren gan 亖 ni [亖:Indicates the number four, pronounced similarly to “death”.]
a23	Substitution of homophonic characters for vulgar expressions (4)	“Ai, sen kou ni dao di yao ma bu yao, yao ma bu yao” [sen kou: bastard, curse word]
a24	Regionalization of honorific vocabulary (27)	zi xie song chui zi guang ting bie ren liao zui zi[song chui zi: a rude nickname]
a25	Diversification of food-related vocabulary (39)	Wo men jia cong xiao jiu si di, di yi guo bi xu shi gei lao ba xia de pian pian la tiao zi.[la tiao zi: type of noodle dish]
a26	Local variation in animal and plant vocabulary (10)	Ji ji gun dao ha di[ji ji: a plant]
a27	Colloquialization of body-related vocabulary (3)	Jin jue ban zi hei liang hei liang di[Jin jiao ban zi: barefoot]
a28	Popularization of everyday vocabulary (22)	Yi lao you xiang ta zu di ni [Yi lao: frequently]
a29	Vividness in action and behavior vocabulary (44)	Yao me qu chong sha yi ge [Cut open another one (watermelon)]
a30	Imagery in emotional state vocabulary (27)	Mian tiao nang nang xi xi zai gei we dai dao [The noodles are overcooked]
a31	Words derived from Shaanxi dialect (1)	Ding…ding dun ha [Ding Ding: stable]
a32	Words from Gansu dialect (6)	Chuan ca ca ku yi da li zhang da di [ca ca ku: a pair of open-crotch pants; yi da li: together]
a33	Words from Ningxia dialect (1)	Zhe shi nei ge hou guo kui ma [guo kui: a baked pasta dish]
a34	Words borrowed from Uyghur (8)	Zhe cai si zhen zheng er zi wa wa! [er zi wa wa: real man]
a35	Words borrowed from Kazakh (2)	Jin tian fa dian mian, zu dian bao er sa ke [bao er sa ke: a type of fried dough]
a36	Idioms in Xinjiang Chinese dialect (12)	Chu jing dao guai ye you shuo yi tian jing zhe teng wan di bu gan zheng shi di yi si [chu jing dao guai: to make a big deal out of nothing]
a37	Colloquial phrases in Xinjiang Chinese dialect (10)	Mo ma da, ga ga di si qing [no problem, it's no big deal.]
a38	Miscellaneous terms in Xinjiang Chinese Dialect (2)	Lao ye zi zhu zai dong bian de er fang zi, mu bei zi, mu ru zi, kang shang pu le ge chai chai zi. ni gei song le ge pi gua zi, gai le ge bi jian zi, dong le ge tui bang zi, gai le ge tui bang zi, dong le ge bi jian zi. gan cui gai le ge xiao du zi, shei zhi dao dong le ge liang tou zi!
a39	Mandarin code-switching (6)	Bu chong yi xia: hai mai si, (All), xue bei er (a little)
a40	Mixed with Cantonese (1)	94 nian shang chu zhong na hui er, qiu tian mei zi mai diao, jia li mai le ge lan se ling mu yi bai, qi shang ba shi ma pao di nie han gua zi kou zi jie kai chao ren dou peng yi yang fei de zhi zhi di, ye si cun li zui liang zai [mei zi: Xinjiang Chinese dialect, wheat] [liang zai: Cantonese, beautiful people]
a41	Mixed with English (3)	Up, wen yi ha you mei you fang yan jiao liu qun ya, zen me jia? [wen yi ha: may I ask]
a42	Simultaneous English translation (4)	Nei ge dian xian gan shang bia di nei ge zhong jin qiu zi [The big money begging son of bia on that telephone pole] [bia: paste]
a43	Mandarin subtitle configuration (9)	Ni ba cong lai mei you ze me ying qi guo [ying qi: the actual pronunciation is “ning bang,” and the actual meaning is “with righteous indignation”.]
a44	Mixed with classical Chinese (2)	Xin jiang hua jiao xue zhi⌈gou zi⌋di mei li [zhi: classical Chinese][gou zi: Xinjiang Chinese dialect, buttocks]
a45	Mixed with internet slang (9)	Qian shui di chu lai xuan sa! [qian shui: internet slang]
a46	A questioning tone (3)	ai, ni ba wo dang bao mi hong ni ma! [ni ma: interjection; How dare you treat me like corn and deceive me]
a47	Expressing advising tone (4)	Ni ba rou jia shang chi sa [sa: admonishing tone]
a48	Expressing declarative tone (3)	Wo nei hui wo song nai zi ne ma [ne ma: the tone of the statement]
a49	Expressing exclamatory tone (15)	ou hou![ou hou: exclamatory tone]
a50	To express a question (8)	Za ban ni sa? [sa: questioning tone]
a51	Expressing impatient tone (1)	ai dui le dui le [dui le dui le: impatient tone]
a52	Pause strategy (2)	Ding…ding dun ha [Stay put.]
a53	Space-based cutoff (4)	Gao shan liu shui hua la la, yi bei xiao jiu wa ha ha.

#### 4.1.2 Axial coding

Axial coding further refines the categories derived from open coding, identifying the relationships among main categories, establishing hierarchical linkages between categories, and verifying these relationships through data. Specifically, it involves an in-depth analysis of sub-categories to distinguish primary and subordinate categories, thereby distilling abstract main categories (Scott, 2004). In this study, six main categories were identified, and the specific relationships between these main categories and their corresponding subcategories are presented in Table 4.

**Table 4:** Axial coding analysis.

Primary category	Subcategory category	Definition (as used by users in Chinese online communities)
Integration of symbols and images into textual expression	Chinese characters	Using Chinese characters to record and express the phonetics and vocabulary of Xinjiang Chinese dialects
	Chinese characters and pictograms	Using Chinese characters and pictorial symbols to express the phonetics and vocabulary of Xinjiang Chinese dialects
	Chinese characters and emoticons	Using Chinese characters and emojis to express the phonetics and vocabulary of Xinjiang Chinese dialects
	Chinese characters and numeric symbols	Using Chinese characters and numeric symbols to express the phonetics and vocabulary of Xinjiang Chinese dialects
	Chinese characters and punctuation marks	Using Chinese characters and punctuation symbols to express the phonetics and vocabulary of Xinjiang Chinese dialects
Practices of transliteration	Transcription using Chinese characters	Using Chinese characters to record the spoken pronunciation of Xinjiang Chinese dialects
	Transcription using Pinyin	Using Pinyin to record the spoken pronunciation of Xinjiang Chinese dialects
	Combined use of Pinyin and Chinese characters	Using Chinese characters and Pinyin to record the spoken pronunciation of Xinjiang Chinese dialects
Fusion of lexical expression	Unique vocabulary	Using vocabulary with Xinjiang Chinese dialect features on Chinese online communication platforms
	Vocabulary consistent with the Northwest region	Using vocabulary consistent with dialects from Northwest China provinces like Shaanxi and Gansu within Chinese online communities, as these terms belong to the Xinjiang Chinese dialect
	Vocabulary borrowed from minority languages	Vocabulary from ethnic languages like Uyghur and Kazakh is used in Chinese online communities, constituting an important component of Xinjiang Chinese dialects
	Idioms of the Xinjiang Chinese Dialect	Using idioms, four-character phrases, set expressions, and proverbs characteristic of Xinjiang Chinese dialects
Distinct rhythm and intonation	Special tonal expressions	Expressing the unique intonation of Xinjiang Chinese dialects through Chinese characters
	Special rhythmic expressions	Conveying the distinctive speech rhythm of Xinjiang Chinese dialects through typographical symbols
Simplification of euphemistic expressions	Euphemistic expressions using Roman letters	Using Roman letters to express euphemisms Employing homophonic archaic characters to convey euphemisms
	Euphemistic expressions using oracle bone script	Using homophonic Chinese characters to express euphemisms
	Euphemistic expressions using other Chinese characters	Blending linguistic resources, including Mandarin Chinese, local dialects, and English
Eclectic language style	Eclectic use of linguistic resources	Mixing formal classical Chinese and Mandarin with colloquial dialects and internet slang
	Blending of formal and informal language	Employ a blend of classical Chinese and Mandarin, alongside colloquial dialects and internet slang.

#### 4.1.3 Selective coding

Selective coding is the process of unifying all categories around a central “core” category, supplemented by descriptive details for categories requiring further elucidation. Through iterative review, comparison, and analysis of raw materials, open coding, axial coding, and analytical memos, this study ultimately subsumed all phenomena under “New media representations of written Xinjiang Chinese dialect and their selection behaviors” as the core category (Table 5).

**Table 5:** Exemplar paradigm relationships of the core category.

Research item	Typical relational structure	The essence of relational structure	Example (Chinese)	Example explanation
The written representation of social media	Integration of symbols and images into verbal expression	The combined use of Chinese characters, images, symbols, etc., constitutes symbolic practices in social media expression.	杨博主, 我们一代人的回忆录, 关灯绳绳子, 是亮点。	Serving as a memoir for our generation. The rope, notably, is the highlight; it is absolutely... (Source: publicly available online corpus, WeChat)
	Practice of transliteration	Utilizing phonetic translations, pinyin transcriptions, and abbreviations of Chinese characters represents symbolic practices in social media expression.	ran 倒是有 ran 就是黏的意思对吧	The morpheme “ran” signifies adhesion. (Source: publicly available online corpus, WeChat)
	Fusion of lexical expression	Employing characteristic words, vocabulary consistent with the Northwest region, borrowed terms from minority languages, and dialect idioms constitute dialect features extracted for social media expression.	木麻哒爆炒椒麻鸡（鼎升嘉园店）	Stir-fried spicy chicken (Dingsheng Jiayuan branch). (Source: publicly available online corpus, Meituan)
	Distinct rhythm and intonation	Distinct rhythms and unique intonations constitute dialect features extracted for social media expression.	想到啥就说撒, 也懒得回头细翻了。如果我有幸能有自己的后人, 我定是要教他说新疆土话的, 传承不能断啊, 其他人一说祖上口里哪儿哪儿的, 我们祖上就是新疆了啊, 这地头就是我们的根啊, 把土话丢了我们还能剩下撒, 啥都么了啊	Spontaneous expressions of dialect, unburdened by overthinking. (Source: publicly available online corpus, Douyin)
	Simplification of euphemistic expression	Employing Roman letters, oracle bone script, and other Chinese characters as euphemistic expressions constitutes an innovative strategy in social media expression.	把人干亖呢	Signifying a state of high engagement or busyness. (Source: publicly available online corpus, Douyin)
	Eclectic language style	The hybrid use of linguistic resources and the blending of highbrow and lowbrow elements represent an innovative strategy in social media expression.	新疆话教学之[钩子]的魅力	This content, presented in the Xinjiang Chinese dialect, explores the allure of the term “pigu” (buttocks) through an instructional lens. (Source: publicly available online corpus, Bilibili)
	Symbols and images integrated into written expression—eclectic language style	The integration of symbols and images into textual expression signifies the fusion of linguistic styles, thereby enabling expression on social media platforms.	拌拉条子吃去攒劲很	This mixed noodle dish is exceptionally palatable. (Source: publicly available online corpus, Douyin)

Centered on the core category, the storylines are summarized as the integration of symbols and images into textual expression, alongside the practice of transliteration, constituting the symbolic practices of new media users. Building on this foundation, users engage in written vernacular expression within new media: on the one hand, they incorporate and display integrated lexical expressions characteristic of Xinjiang Chinese dialect, along with its distinctive rhythmic intonation; on the other hand, they employ pragmatic strategies of euphemistic expression and stylistic hybridity. Following the overarching pathway of “dialectal features—innovative strategies—symbolic practices,” this study has constructed a model of “Written Vernacularization of Xinjiang Chinese Dialect in New Media and Its Selection Behaviors” (Figure 4).

**Figure 4:**
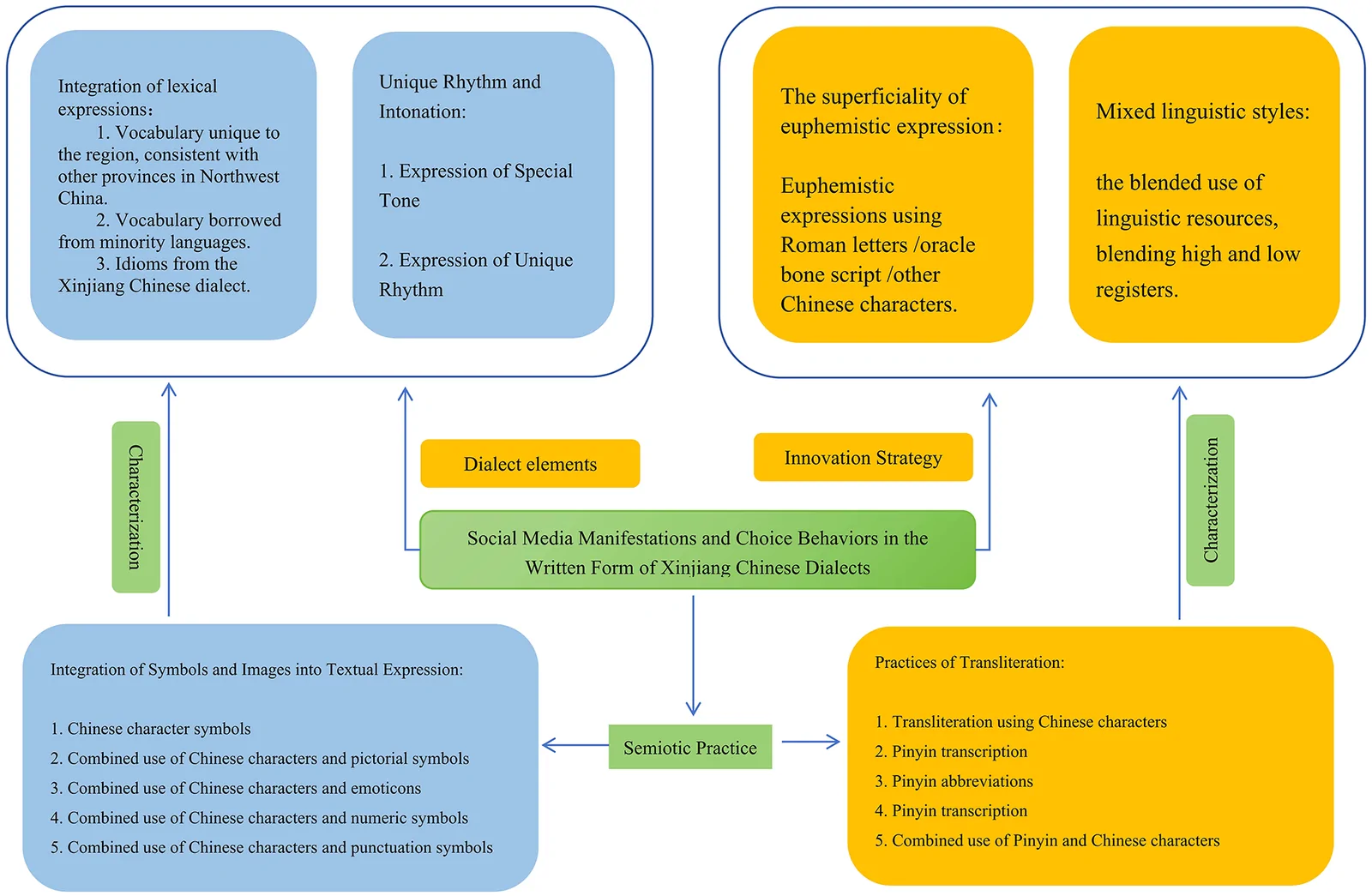
Model of social media representation of written Xinjiang Chinese dialects and associated choice behaviors. This figure was developed by the authors based on the data and analytical framework of the present study.

### 4.2 Core findings of the qualitative research

The qualitative analysis of this study primarily centers on the practices of digital dialect expression within the Xinjiang social media context. Results from open coding indicate that the relevant linguistic forms mainly involve features such as dialectal character usage, homophone substitution, transliterated expressions, intensification of tone, code-switching, and emoji combinations. These expression forms exhibit pronounced contextualization and interactivity across different interaction scenarios, revealing that digital dialect in online communication not only serves as a tool for conveying meaning but also constitutes a motivation-driven communicative strategy. Users consciously select or adjust dialect expressions in various social contexts to achieve specific communicative goals and emotional expression needs.

Further axial coding demonstrates that different types of digital dialect expressions exhibit significant differences in interactive functions, reflecting multi-layered psychological processing experiences. For instance, linguistic forms featuring repeated characters, tonal intensification, or emotionalized expressions emerge more frequently in highly interactive and participatory social contexts. Such expressions often function to evoke emotional responses from the audience, enhancing the emotional tension of the text to boost interactive engagement. In contrast, linguistic practices involving region-specific vocabulary, local appellations, and in-group expressions more strongly reflect social familiarity and intra-group communication characteristics. Their core function lies in evoking resonance within the group through shared linguistic resources, thereby reinforcing identity recognition and a sense of belonging. Moreover, some dialect expressions exhibit clear contextual adaptability, with users flexibly adjusting their expressive strategies in response to changes in the interaction partner, communicative atmosphere, and platform context. This process reflects individuals' capacity for proactive self-regulation in social interactions—namely, strategically adapting their linguistic behavior after perceiving the differing demands of various social situations.

The practice of digital dialect expression in Xinjiang is not entirely an unconscious habitual behavior but is embedded within a certain oriented cognitive framework. When deciding whether to employ dialect expressions, which form to adopt, and the depth of expression, users are often influenced by multiple factors, including audience receptivity, platform normative constraints, and potential social evaluations. This careful assessment of the social context itself constitutes a risk perception process—users must weigh dialect against communicative effectiveness, balancing the assertion of regional identity with the avoidance of communication barriers. Simultaneously, the use of digital dialect also exhibits a driving characteristic, whereby specific social motivations (e.g., seeking recognition, expressing intimacy, or constructing distinction) directly prompt individuals to select corresponding dialect expression forms, making linguistic practice a bridge between motivation and behavior.

In summary, digital dialect expressions in Xinjiang not only manifest as differentiated practices at the linguistic form level but also exhibit multiple interactional features, such as emotional arousal, interaction enhancement, identity marking, group resonance, motivation-driven behavior, and self-regulation. At the same time, they are modulated by an oriented cognitive framework and risk perception. These empirical characteristics further provide an empirical foundation for the construction of psychological dimensions and the design of experimental stimuli in subsequent quantitative research.

#### 4.2.1 Dialectal practices of emotional expression and interactive contagion

In the dialectal writing on new media platforms, images and emoji stickers function as emotion-mediating processes, compensating for the limitations of plain text in conveying affect through visual means, thereby giving rise to a psychological writing mode oriented toward emotional arousal and affective resonance. (1) Precise cueing of individual emotional arousal. The affective connotations carried by dialectal terms are often prone to misinterpretation due to regional differences; by contrast, emoji stickers—through visual symbols such as exaggerated facial expressions and dynamic gestures—render abstract emotions concrete, enabling more intuitive and accurate transmission of affect. For example, the Xinjiang Chinese dialect term “Lao Dao” (meaning “awesome” or “impressive”), when paired with a GIF of “Uncle Naan” repeatedly giving a thumbs-up, instantly intensifies the emotion of praise or self-boasting, reducing the potential comprehension bias that plain text may entail. (2) Can be interpreted as triggering collective affective resonance. Social categorization theory (Banton, 2011) posits that people often delineate “in-groups” through shared cultural symbols (e.g., dialects) and reinforce their sense of identity and belonging. In new media interactions, the use of dialect and its accompanying emoji stickers implicitly marks group identity, exhibiting a tendency toward specific emotional expression and identity recognition. For instance, a dialectal comment under a Xinjiang Chinese dialect video may garner dozens of likes precisely because it simultaneously cues individual emotional arousal and serves as a group symbol linked to collective resonance. Thus, the synergistic use of dialect and visual symbols constitutes a complete psychological chain from cueing individual emotional arousal to strengthening the interactive atmosphere. This not only enhances the affective expressiveness of dialectal writing but also reinforces intra-community interactive cohesion, underscoring the pivotal role of emotion-driven dynamics in the digital dissemination of dialects.

#### 4.2.2 Dialect practices as identity markers and group familiarity

Dialect writing in new media is not a direct transcription of offline spoken language; rather, it constitutes an identity practice grounded in technological plasticity, in which symbolic strategies transform local vernaculars into visual identity markers, thereby facilitating social recognition and group positioning within cyberspace. On the one hand, lexical hybridity emerges as a salient feature. The lexical system of Xinjiang Chinese dialect exhibits pronounced hybrid characteristics, serving as a core symbol that distinguishes it from other dialects and underscores local identity, reflecting the territorialization of dialect features (Poon, 2017). This is manifested in the following: (1) the use of specific lexemes reflecting local cuisine, geography, and worldview, such as LaTiaoZi (hand-pulled noodles), DaoTiaoLing (Knife Ridge), and Chuizi (hammer); (2) retention of lexemes cognate with Northwestern Chinese dialects, such as GuoKui (flatbread) and LaoHan (old man), reflecting historical lineage; (3) incorporation of loanwords from minority languages, e.g., Uyghur PiYaZi (onion) and Kazakh BaoErSaKe (fried dough); and (4) the use of idioms imbued with local character, such as GaGaDeShiQing (a trivial matter). Rather than being diluted within the cross-regional, fluid network space, these lexemes have instead reinforced the “stickiness” of the language, serving as vital resources for sustaining regional identity.

On the other hand, the textual representation of prosody and intonation constitutes a parallel strategy. Users creatively transcribe sound features that cannot be directly represented in writing, thereby conveying the distinctive tone and rhythm of the dialect. For example, modal particles such as Sha and Nima are used to express modality; in phrases like Ding…ding dunha, ellipsis and reduplication simulate the length and repetition of syllables in speech. Such textualized prosody, even when read silently, activates shared phonological memories, enabling users in distant locations to decode messages based on common cultural backgrounds, thus implying regional identity and emotional bonds within virtual space.

In summary, analysis of lexical hybridity reconstructs the symbolic foundation of identity, while prosody and intonation infuse sonic identity and affective bonds. Together, they enable Xinjiang regional identity to maintain strong cultural stickiness within the “smooth space” of the internet, which can be interpreted not only as a challenge to the dominant discourse order of Standard Mandarin but also as a continuous driver of dialect-based online community formation and identity reproduction.

#### 4.2.3 Dialect practices for social participation and expressive enhancement

In the new media environment, the written representation of Xinjiang Chinese dialects constitutes a motivationally anchored symbolic practice. It reflects users' psychological processes of reinforcing interaction and community identity through multimodal resources, driven jointly by the need for self-expression and the willingness to participate.

(1) Integrated Use of Multimodal Symbols. Writing is a representation of speech—a representation of words uttered by a speaker or imagined as uttered. A writing system typically comprises only twenty to forty characters, and when necessary, one system can be substituted for another. The Chinese character writing system is applicable to all dialects and has undergone prolonged, diachronic continuity (Lam and Lau, 2017). Thus, the Xinjiang Chinese dialectal expression meaning “impressive” (Lihai) can be written as “LaoDao”, while the place name “Qitai” can be rendered in traditional characters as “QiTai”. Beyond the exclusive use of Chinese characters, users also combine characters with images, emoticons (e.g., kaomoji, stickers), and textual transcriptions accompanied by pictures. Furthermore, characters are integrated with digital symbols and punctuation marks. For example, “SanShiLiHei” represents a four-character phrase in Xinjiang Chinese dialect; its standard written form is “SanShiLiHei” (lit. “the night of the thirtieth”), referring to the final night of the twelfth lunar month. In “Na = Ta”, the equal sign indicates synonymy: the left side is the Xinjiang Chinese dialectal form, and the right side is the Mandarin interpretation. The spatiotemporal compression and symbolic recombination functions of digital media (Deng, 2025) greatly facilitate the real-time dissemination and sharing of these localized cultural symbols within online communities, thereby enhancing the visibility and interactive potential of dialectal expressions. These hybrid symbols not only create a distinctive local online style, strengthening users' identity and expressive motivation, but also, at a deeper level, transform localized cultural symbols into nostalgic scenes imbued with ethnic imagery, driving the digital reproduction of collective memory (Hu and Deng, 2025).
(2) Diverse Strategies for Phonetic Transcription. To accurately capture and disseminate the unique pronunciations of the dialect, users primarily employ three transcription strategies: (a) Transcription via Chinese characters. Chinese characters are morphosyllabic graphs; the morphemes they represent constitute sound–meaning composites (Liu, 2025). For instance, in “XingLeSa”, many Xinjiang Chinese dialect sentences contain the sentence-final particle /sa/, and users approximately represent this dialectal pronunciation with the character “Sa”. (b) Transcription via pinyin. Pinyin is a visual symbol system, but it is fundamentally rooted in the auditory channel—essentially a transcription of phonetic signals (Zhang, 2023). For example, “nia”: in Standard Mandarin phonology, the initial /n/ cannot combine with the final /ia/. Nonetheless, users transcribe the old-style pronunciation of “Ta/Ta” (he/she) in the Xinjiang Chinese dialect by applying the Standard Mandarin phonemic inventory. (c) Combined use of pinyin and characters, integrating the two previous methods. For example, “Ni fo sa di ne” means “What are you saying?” Here, users transcribe the pronunciation of “Shuo” (say) with the pinyin “fo”. In Xinjiang, there is a phenomenon of homophony between “Shu Fu” (comfortable) and another form—specifically, in some counties and cities, syllables originally taking the Standard Mandarin initial /ʂ/ with a closed mouth (he kou hu) are pronounced with initial /f/ with an open mouth (kai kou hu). The character “sa” follows the same pattern as explained above.

In summary, whether through multimodal integration or phonetic transcription, the fundamental motivation is to transform dialects from localized oral speech into dynamic symbolic resources that circulate within broader digital spaces. By selecting standard written symbols for transcription, users lower the threshold for comprehension, effectively constructing a participatory cultural space that encourages interaction, imitation, and creative reproduction. This process not only satisfies users' individual expressive needs but also, driven by participation, continuously activates Xinjiang Chinese dialects in the new media context, endowing them with renewed vitality.

#### 4.2.4 Dialect practices of situational adaptation and expressive adjustment

Within the regulatory environment of new media platforms, dialect writing is simultaneously shaped by platform norms and the potential risk of conflict. Users consequently develop risk-perception-based self-regulatory processes to balance expressive demands against communicative constraints.

(1) Surface-Level Euphemistic Expressions. To mitigate risks associated with sensitive or taboo content (e.g., insults, death, derogatory epithets), users strategically mobilize symbolic resources such as Roman letters, oracle bone script, and other Chinese characters, thereby constructing surface-level euphemisms. For instance, “b” is employed to insinuate a certain insult; the oracle bone character “亖” (homophonous with “Si” [death]) substitutes the character “Si”; and “Sen kou” (homophonous with “sheng kou” [brute]) replaces the original term. These practices integrate the user's linguistic acquisition experience with digital risk awareness, functioning as a self-regulatory mechanism for evading expressive risks.
(2) Hybrid Use of Linguistic Resources. Users mix multiple linguistic resources across lexical and stylistic dimensions, resulting in a composite style. For example, Cantonese “Liang zai” (handsome guy), English “up”, or a title blending the Classical Chinese particle “Zhi” with the dialect term “Gou zi” (buttocks). This hybridity effectively recontextualizes linguistic resources from disparate contexts into the new media platform. Although it often draws on “curtailed language skills” and may lack grammatical conformity, it effectively constructs regional identity, affinity, and a humorous effect, demonstrating the user's active pragmatic adaptation to a heterogeneous audience.

### 4.3 Summary of qualitative analysis

Thematic analysis of 965 social media utterances in the Xinjiang Chinese dialect reveals that dialectal writing in digital interactional contexts functions not only as a marker of linguistic variation but also as a vehicle for emotional expressiveness and interactional orientation.

In the open coding phase, several initial concepts were identified, including dialectal lexical usage, phonological transcription, emotionally intensified expressions, group-specific appellations, interactional tone, code-mixing, coordinated use of emoji, and local expressions. Subsequent axial coding indicated that these linguistic practices cluster around two relatively stable interactional dimensions. The first involves the use of dialectal expressions to intensify the emotional atmosphere and expressive force; the second entails the deployment of localized linguistic forms to establish a sense of in-group familiarity and interactional proximity.

Specifically, a substantial portion of the data exhibits marked affective intensification. For instance, users tend to employ regionally colored interjections, reduplicative forms, and phonetic transcriptions, which are associated with emotional contagion and vividness of expression. In highly interactive comment threads, dialectal expressions often co-occur with exaggerated intonation, humorous phrasing, and colloquial narration, thereby endowing the language with heightened emotional immediacy.

At the same time, dialectal expressions frequently appear in interactional contexts characterized by group identification. Users establish an “one of us” interactive atmosphere through localized appellations, regional vocabulary, and shared expressions. Such expressions not only reflect familiarity with regional culture but also exhibit a strong tendency toward social affinity at the interactional level. For example, in comment replies, playful banter, and collective emotional expression, dialectal forms tend to generate extended interactional chains.

Furthermore, the analysis reveals that certain linguistic practices simultaneously fulfill both emotional expression and social identity functions (Goldenberg and Gross, 2020; Li and Shen, 2025). For instance, some expressions with pronounced local color are associated both with higher ratings of emotional contagion and with a sense of familiarity and group belonging during interaction. This intertwining suggests that dialectal expressions on social media are not merely tools for information transmission but rather interactional resources imbued with both emotional and social dimensions.

The findings of Study 1 indicate that the Xinjiang Chinese dialect in digital communication contexts exhibits two core trends: first, it is associated with emotional contagion and expressive intensity; second, it strengthens social proximity and group identity during interaction. These results provide an empirical foundation for the construction of dimensions and the development of scales in subsequent quantitative research.

### 4.4 Correspondence between qualitative themes and quantitative measurement dimensions

Study 1 (qualitative) employed open coding, axial coding, and thematic induction on dialectal interaction texts from Xinjiang social media, yielding 53 initial coding categories and 6 core themes. These themes primarily pertained to emotional expression, local familiarity, interactive participation, group belonging, language affinity, and social transmission tendency. Collectively, these themes exhibited strong interactivity and contextual relevance, indicating that digital dialect expression not only possesses linguistic distinctiveness but also embodies multiple experiential meanings, such as emotional arousal, relationship construction, and social interaction.

In Study 2 (quantitative), these themes provided an empirical foundation for stimulus selection, evaluation dimension specification, and scale item design. Themes related to “emotional expression”, “expressive appeal”, and “authenticity” primarily corresponded to the subsequent evaluation dimension of emotional contagion and expression intensity. Meanwhile, themes involving “group belonging”, “localness”, “interactive participation”, and “social transmission tendency” were more reflected in dimensions associated with social identity and interaction tendency. Exploratory factor analysis further revealed that participants' perceptions of digital dialect expression did not form multiple fully independent constructs but rather coalesced into two interrelated core experiential dimensions: “emotional contagion and expression intensity” and “social identity and interaction tendency”. This finding indicates a high degree of consistency between the interactive themes identified in Study 1 and the experiential dimensions in Study 2. It also suggests a certain degree of interweaving and overlap between emotional experience and social relational perception in digital dialect interaction (Table 6).

**Table 6:** Correspondence of themes between qualitative and quantitative research.

Study 1 (qualitative)		Study 2 (quantitative)	
Qualitative themes	Keywords	Evaluation dimensions	EFA attribution experience dimension
Emotional expression and persuasiveness	More emotionally resonant/more dynamic typical coded content/more atmospheric	Intensity of emotion, infectiousness, emotional resonance	Emotional Contagion and Expressive Intensity
Local familiarity	Speaks like a local/has a stronger Xinjiang accent	Locality, linguistic familiarity	Social identity and interaction orientation
Sense of group belonging	Feels like one of our own/creates a stronger sense of community	Group belonging, identity proximity	Social identity and interaction orientation
Tendency toward social interaction	More likely to prompt replies/easier to interact	Will to interact, tendency to use social media	Social identity and interaction orientation
Language communicability	Suitable for comment sections/better suited for social media expression	Social media usage tendency	Social identity and interaction orientation
Sense of situational authenticity	More authentic/more down-to-earth	Authenticity of expression, emotional presence	Emotional contagion and expressive intensity

### 4.5 Core findings of the quantitative study

#### 4.5.1 Descriptive statistical results

To compare the differences between Xinjiang Chinese dialect expressions and Mandarin expressions across various perceptual dimensions, a descriptive statistical analysis was first conducted on each item. The overall results indicate that participants generally rated Xinjiang Chinese dialect expressions more highly than Mandarin expressions on subjective evaluations. In particular, items related to “emotional contagion”, “vividness of expression”, “willingness to interact”, and “ingroup familiarity” showed higher mean scores for dialect expressions than for the Mandarin condition. In terms of distributional characteristics, the standard deviations for all variables fell within a reasonable range, with no pronounced extreme skewness. Mean scores under the dialect condition were generally higher than those under the Mandarin condition, preliminarily suggesting that dialect expressions may possess stronger emotional expressiveness and interactive appeal in the context of social media interaction. Ethnicity information in this study was primarily used to describe the basic sample structure (Table 7). Given the relatively limited sample sizes for certain ethnic subgroups and the lack of sufficiently stable structured subdivisions in the existing data, no formal cross-ethnic statistical comparisons were conducted. Consequently, the current findings are more applicable to general empirical observations within specific digital dialect interaction contexts and should not be directly generalized to all ethnic groups. Furthermore, the results of subsequent exploratory factor analysis (EFA) indicated that the scale structure did not form the originally hypothesized set of fully independent constructs. Therefore, the subsequent statistical analyses were based on the empirically derived latent factor structure, focusing on two core experiential dimensions: F1 (Emotional Contagion and Expressive Intensity) and F2 (Social Identity and Interactional Orientation).

**Table 7:** Basic demographic information.

Variables	Options	Frequency	Percentage (%)
Ethnicity	Kazakh	8	5.37%
	Hui	12	8.05%
	Tajik	1	0.67%
	Kyrgyz	1	0.67%
	Han	102	68.46%
	Manchu	1	0.67%
	Uyghur	23	15.44%
	Zang	1	0.67%
Gender	Male	93	62.42%
	Female	56	37.58%
Education	High school or below	2	1.34%
	Associate's/Bachelor's degree	142	95.30%
	Graduate	5	3.36%
Total		149	100.00%

#### 4.5.2 Results of the EFA

To examine the latent structure of the self-developed scale, an exploratory factor analysis (EFA) was conducted on the nine Likert-scale items, preceded by an assessment of data adequacy. The KMO measure yielded a value of 0.923, indicating substantial common variance among the variables and supporting the suitability of factor analysis (Table 8). Bartlett's test of sphericity was significant (χ^2^ = 4391.123, *p* < 0.001), further confirming the presence of strong inter-variable correlations necessary for factor extraction (Table 8). Factor extraction employed principal axis factoring with oblique rotation (Table 9). Two primary latent factors were identified: factor 1 (Emotional Contagion and Expressive Intensity) and factor 2 (Social Identity and Interactional Tendency), jointly accounting for 78.282% of the total variance (Table 10). After rotation, Factor 1 explained 39.590% of the variance, and Factor 2 accounted for 38.692%, with the two factors contributing comparably, thereby indicating a stable two-dimensional structure for the scale.

**Table 8:** KMO and Bartlett's tests.

Testing method	KMO	0.923
Bartlett's test of sphericity	Approximate chi-square	4,391.123
	df	153
	*p*	0.000

**Table 9:** Rotated factor loading matrix.

Scale items	Scale content options	Factor loadings		Communality
		F1	F2	
Q7–1(A)	This expression makes me feel more emotionally charged.	0.252	0.806	0.712
	This expression is more vivid/infectious.	0.674	0.608	0.824
	This expression is more likely to evoke emotional resonance.	0.710	0.596	0.859
	This expression makes me feel more “local”.	0.735	0.535	0.827
	This expression better reflects a sense of belonging to a group.	0.900	0.341	0.927
	This expression sounds more like the way “one of us” speaks.	0.884	0.341	0.898
	I'd rather reply this way.	0.909	0.333	0.937
	This expression is more likely to spark interaction.	0.906	0.327	0.929
	I'm more likely to use this expression on social media.	0.891	0.245	0.854
Q8–2(A)	This expression makes me feel more emotionally charged.	0.224	0.759	0.626
Q9–3(A)	This expression makes me feel more emotionally charged.	0.319	0.827	0.786
Q10–4(A)	This expression makes me feel more emotionally charged.	0.266	0.763	0.653
Q11–5(A)	This expression makes me feel more emotionally charged.	0.274	0.794	0.706
Q12–6(A)	This expression makes me feel more emotionally charged.	0.448	0.735	0.741
Q13–7(A)	This expression makes me feel more emotionally charged.	0.472	0.593	0.574
Q14–8(A)	This expression makes me feel more emotionally charged.	0.443	0.770	0.788
Q15–9(A)	This expression makes me feel more emotionally charged.	0.373	0.686	0.609
Q16–10(A)	This expression makes me feel more emotionally charged.	0.689	0.605	0.842

**Table 10:** Explanation of total variance.

Factor	Initial eigenvalues			Extraction of sum of squares of loads			Sum of squares of rotational loads		
	Eigenvalues	Variance explained (%)	Cumulative (%)	Eigenvalues	Variance explained (%)	Cumulative (%)	Eigenvalues	Variance explained (%)	cumulative (%)
F1	12.377	68.763	68.763	12.377	68.763	68.763	7.126	39.590	39.590
F2	1.713	9.519	78.282	1.713	9.519	78.282	6.965	38.692	78.282

Based on item loadings, Factor 1 primarily reflected emotional contagion and expressive tension arising from linguistic expression. Items such as “more intense emotions”, “more infectious”, and “more likely to trigger emotional resonance” exhibited high loadings on this factor; accordingly, it was labeled “Emotional Contagion and Expressive Intensity”. Factor 2 is centered on group belonging, interactional tendencies, and willingness to use language for social purposes. Items including “better reflects group belonging”, “a way of speaking more like one's own people”, “more willing to use this expression in replies”, and “more likely to co-occur with interactional tendencies” loaded highly on this factor; thus, it was named “Social Identity and Interactional Tendency”. Several items displayed cross-loadings on both factors, notably “more vivid/infectious”, “more likely to be accompanied by emotional resonance experiences”, and “more locally grounded”. This pattern suggests that emotional contagion and social identity are not entirely separable in dialect-mediated interaction experiences but are instead strongly interrelated. In other words, the emotional experiences elicited by dialectal expression are frequently accompanied by a sense of group familiarity and interactional closeness. Overall, the EFA results did not support the originally hypothesized four fully independent dimensions; rather, they converged on two core, empirically interrelated experiential dimensions—F1 (Emotional Contagion and Expressive Intensity) and F2 (Social Identity and Interactional Tendency). Consequently, subsequent analyses will employ these empirically derived factors for further statistical comparisons and result interpretation.

#### 4.5.3 Scale reliability and item consistency analysis

To further examine the internal consistency of the two empirically derived factors, reliability analyses were conducted on the F1 and F2 subscales, respectively (Table 11). The results showed a Cronbach's α coefficient of 0.971 for the F1 subscale, with corrected item-total correlations ranging from 0.918 to 0.956. Removal of any single item did not result in a marked improvement in α (α = 0.943–0.970), indicating that this subscale possesses high internal consistency. For the F2 subscale, the Cronbach's α coefficient was 0.965, demonstrating high internal consistency. The corrected item-total correlations for all items ranged from 0.841 to 0.956, confirming that each item maintains a strong association with the overall dimension. Further deletion analysis revealed that removal of any individual item did not substantially increase the α coefficient (α = 0.952–0.968), suggesting that all items contribute stably to the dimension.

**Table 11:** Scale reliability and item consistency analysis.

Factor	items	Sample size	corrected items - total score correlation	α coefficient after item removal	dimension α coefficient
F1	This expression makes me feel more emotionally charged	149	0.936	0.957	0.971
	This expression is more vivid/infectious.	149	0.918	0.970	
	This expression is more likely to evoke emotional resonance.	149	0.956	0.943	
F2	This expression better reflects a sense of belonging to a group.	149	0.900	0.958	0.965
	This expression sounds more like the way “one of us” speaks.	149	0.924	0.953	
	I'd rather reply this way.	149	0.917	0.955	
	This expression is more likely to spark interaction.	149	0.933	0.952	
	I'm more likely to use this expression on social media.	149	0.841	0.968	

Both subscales exhibited high levels of internal consistency. On the one hand, this indicates that the scale items reliably reflect the corresponding experiential dimensions. On the other hand, it also suggests a certain degree of content proximity among some items. Considering the cross-loading phenomena observed in the exploratory factor analysis, the present study posits that emotional contagion, familiarity, and social proximity within social media dialect interactions may inherently be highly interwoven, such that the associated psychological experiences may not be fully separable into independent processes. Given that this study is primarily oriented toward the development of an exploratory, context-specific measurement tool, and that the sample size is relatively limited, the current findings should be interpreted as preliminary structural validation. Future research should further examine the scale structure in larger samples and across diverse contexts, and employ confirmatory factor analysis (CFA) along with indices such as McDonald's ω to conduct more rigorous psychometric validation.

#### 4.5.4 Comparative analysis of different language forms on two core dimensions

Based on the exploratory factor analysis results, this study further calculated the scores on the F1 and F2 scales for different language forms (Table 12). For items constituting the F1 dimension, the mean score was 3.247 (SD = 1.054), while for the F2 dimension, the mean score was 3.365 (SD = 1.008). A repeated measures analysis of variance (ANOVA) was then conducted to compare the score differences between the two dimensions (Table 13), revealing a significant difference between the two perception scores, *F* (1, 148) = 4.786, *p* = 0.030, partial η^2^ = 0.031. Although the effect size was relatively small, the result indicates that participants' psychological responses on the two dimensions exhibited a certain degree of differentiation. Subsequent paired comparisons (Table 14) showed that scores on the F1 dimension were significantly higher than those on the F2 dimension, with a mean difference of 0.118, *t* (148) = 2.188, *p* = 0.030. This suggests that, relative to social belonging and interaction tendency, participants are more likely to form positive perceptions of dialectal expressions first through emotional contagion and expressive vividness. The finding implies that the advantage of dialect expressions in digital interactions may initially manifest as a communicative resource characterized by emotional tension and immediacy of expression, whereas social identity and interaction proximity operate more as ancillary social interaction experiences. In other words, the influence of dialect writing on users does not rely entirely on explicit group identity, but may first activate interactive perception through emotional contagion and expressive vividness. Overall, the role of Xinjiang Chinese dialects in social media contexts extends beyond serving as markers of local identity; rather, it likely enhances users' social experience and interaction tendencies by intensifying the emotional appeal and interactive vividness of expression.

**Table 12:** Comparison of scores on the F1 and F2 scales.

Factor	Time/conditions	*M*	SD	SE	*N*
F1	Emotional contagion/intensity of expression	3.365	1.008	0.083	149
F2	Group identification/social use tendency	3.247	1.054	0.086	149

**Table 13:** Repeated measures ANOVA.

Source of variation	SS	df	MS	*F*	*p*	*ηp* ^2^
Time	1.032	1	1.032	4.786	0.030^*^	0.031
Error (time)	31.904	148	0.216			

^*^p < 0.05.

**Table 14:** Pairwise comparisons (paired *t*-test).

Comparison	Mean difference	SE	*t*	df	*p* (correction)
Group identification/social use tendency (F2)-emotional contagion/intensity of expression (F1)	−0.118	0.054	−2.188	148	0.030^*^

The calibration method is Bonferroni; ^*^p < 0.05.

#### 4.5.5 Repeated-measures analysis under paired-stimulus conditions

To further address the paired-comparison logic embedded in the experimental design, this study reconstructed a within-subject repeated-measures analytical framework based on 10 pairs of dialect–Mandarin stimuli. Given that all participants completed ratings for both the dialect and Mandarin versions across all 10 stimulus pairs, the experimental design was explicitly defined as a 2 (language variety: Xinjiang Chinese dialect vs. Mandarin) × 10 (stimulus pairs) within-subjects design. Comparative analyses were conducted separately along two core experiential dimensions: emotional contagion and expression intensity, and social identification and interaction tendency.

The results indicated that, at the aggregate level, participants rated Xinjiang Chinese dialect expressions consistently higher than their Mandarin counterparts, with the difference being more pronounced on the dimensions of emotional contagion and expression intensity. Further repeated-measures analysis revealed a significant difference between the two core dimensions, *F*_(1, 148)_ = 4.786, *p* =0.030, partial η^2^ = 0.031. Although the overall effect size was relatively small, the findings suggest that participants' positive perception of dialect expressions is primarily manifested in terms of emotional contagion, vividness, and expressive tension, whereas social identification and interaction tendency emerge as a secondary yet still meaningful experiential dimension.

In addition, some degree of variability was observed across different stimulus pairs. This indicates that the psychological effects of dialect expression are not entirely fixed but may be jointly influenced by specific content, contextual familiarity, and the intensity of local cultural coloring. Accordingly, this study posits that dialect experiences in digital social contexts exhibit a certain degree of contextual dependency, rather than constituting a single, stable language-evaluation differential. Because some Xinjiang Chinese dialect expressions lack fully equivalent Mandarin counterparts in the strict sense, the paired comparisons in this study were primarily established on the basis of functional semantic proximity rather than strict word-for-word translational equivalence. Given this characteristic, the study focuses on participants' overall experiential perceptual differences elicited by the two language varieties, rather than on narrowly linguistic full semantic equivalence. Taken together, these results further support the study's interpretation of a “two-dimensional experiential structure” for digital dialect interaction, namely that the role of the Xinjiang Chinese dialect on social media is not limited to serving as a marker of local identity but, more fundamentally, represents an expressive resource associated with heightened emotional contagion and interactive vividness.

#### 4.5.6 Exploratory comparative analysis of demographic variables and experiential dimensions

To further examine the relationship between demographic variables and digital dialect experience, an exploratory correlation analysis, independent-samples *t*-test, and one-way analysis of variance (ANOVA) were conducted based on two core experiential dimensions, F1 and F2.

(1) Correlation analysis results

The correlation analysis results (Table 15) revealed a significant strong positive correlation between F1 and F2 (Pearson r = 0.795, *p* < 0.001; Spearman ρ = 0.755, *p* < 0.001), indicating that although the two experiential dimensions differ in emphasis, they exhibit a high degree of overall consistency.

**Table 15:** Correlation analysis of F1 and F2 with dialect familiarity and frequency.

Variables	Mean	SD	F1	F2	Dialect familiarity	Frequency of dialect use
F1	3.247	1.054	1			
F2	3.365	1.008	0.755^**^	1		
Dialect familiarity	2.987	1.241	0.144	0.241^**^	1	
Frequency of dialect use	2.342	1.196	0.206^*^	0.261^**^	0.443^**^	1

^*^p < 0.05, ^**^p < 0.01.

Age showed a significant but weak positive correlation with scores on both F1 and F2. Specifically, age was significantly weakly positively correlated with the F1 dimension (r = 0.240, *p* = 0.004) and with the F2 dimension (r = 0.246, *p* = 0.003). This finding suggests that older participants tend to exhibit slightly higher positive evaluative inclinations toward the relevant expressions overall. Furthermore, dialect familiarity demonstrated a significant weak positive correlation with F2 (ρ = 0.241, *p* = 0.003), but its correlation with the F1 dimension did not reach statistical significance (ρ = 0.144, *p* = 0.080) (Table 16). In contrast, dialect use frequency exhibited a significant weak positive correlation with both dimensions, with a relatively stronger association with the F2 dimension (ρ = 0.261, *p* = 0.001). Additionally, there was a significant positive correlation between dialect familiarity and dialect use frequency (ρ = 0.443, *p* < 0.001), suggesting a high degree of concordance between these two variables at the experiential level. Overall, the digital dialect experience is associated not only with linguistic expressions themselves but also, to some extent, with individual age, dialect exposure, and daily usage habits. However, the correlation effects are generally weak; therefore, these results should be interpreted as exploratory findings.

**Table 16:** Correlation analysis results of F1 and F2 with age.

Variables	Mean	SD	F1	F2	Age
F1	3.247	1.061	1		
F2	3.365	1.005	0.795^**^	1	
Age	20.841	7.781	0.240^**^	0.246^**^	1

^**^p < 0.01.

(2) Gender differences analysis

Results from the independent samples *t*-test (Table 17) indicate that gender differences in neither of the two experiential dimensions reached statistical significance. Scores on Dimension F1 were comparable between male and female participants (males: *M* = 3.254, SD = 1.064; females: *M* = 3.236, SD = 1.046; *t* = 0.101, *p* = 0.920). Similarly, differences on Dimension F2 were not statistically significant (males: *M* = 3.366, SD = 1.024; females: *M* = 3.363, SD = 0.989; *t* = 0.015, *p* = 0.988). These results demonstrate that the observed digital dialect experience does not exhibit pronounced gender-related variation.

**Table 17:** Analysis of dimensions F1 and F2 by gender.

Factor	Gender (mean ± standard deviation)		*t*	*p*
	Male (*n* = 93)	Female (*n* = 56)		
F1	3.254 ± 1.064	3.236 ± 1.046	0.101	0.920
F2	3.363 ± 1.024	3.363 ± 0.989	0.015	0.988

(3) Analysis of educational attainment differences

The results of one-way ANOVA (Table 18) indicate that educational attainment exerts a statistically significant effect on Dimension F1 (*F* = 3.430, *p* = 0.035), whereas no significant effect was observed on Dimension F2 (*F* = 2.910, *p* = 0.058). *Post-hoc* comparisons reveal that participants with post-graduate education scored significantly higher on Dimension F2 than those with an associate or bachelor's degree (MD = 1.200, *p* = 0.012), with no other between-group differences reaching statistical significance. Overall, participants with higher educational backgrounds tend to exhibit relatively more positive evaluations on the dimensions of social identification and interactional orientation in digital dialect engagement (Table 19). It should be noted, however, that the sample sizes for certain educational levels are relatively limited, and the present demographic analyses are primarily exploratory in nature. Overly substantive inferences should be avoided, and future research should employ larger and more balanced sample structures to further validate the observed differences.

**Table 18:** Analysis of differences between F1 and F2 students and educational attainment level.

Factor	Educational background (Mean ± SD)			*F*	*p*
	High school or below (*n* = 2)	Associate's/bachelor's degree (*n* = 142)	Graduate (*n* = 5)		
F1	3.70 ± 0.99^ab^	3.20 ± 1.05^b^	4.40 ± 0.57^a^	3.430	0.035^*^
F2	4.00 ± 1.41	3.32 ± 1.00	4.33 ± 0.71	2.910	0.058

[^*^] represents a significance marker, [^**a**^] and [^**b**^] are post-hoc comparison grouping markers, and [^**ab**^] is a transitional marker of double insignificance.

**Table 19:** Analysis of differences between F1 students and students with different educational backgrounds.

Factor	Educational 1	Educational 2	Mean	Mean	Difference	Standard error	*p*	Cohen's d
F1	High school or below	Associate's/bachelor's degree	3.700	3.200	0.500	0.738	0.499	0.478
	High school or below	Graduate	3.700	4.400	−0.700	0.867	0.421	−1.041
	Associate's/Bachelor's degree	Graduate	3.200	4.400	−1.200	0.472	0.012^*^	−1.157

[^*^] represents a significance marker.

## 5 Discussion

### 5.1 Core experiential structures in digital dialect interaction

This study investigates the expressive practices of the Xinjiang Chinese dialect in social media contexts, integrating qualitative textual analysis with experimental perceptual evaluation to examine the experiential structures and interactional characteristics of digital dialect interaction. The results of exploratory factor analysis (EFA) indicate that participants' perceptions of dialectal expressions do not align with the initially hypothesized multiple independent constructs but are instead more appropriately captured by two interrelated experiential dimensions: “emotional contagion and expression intensity” and “social identity and interaction inclination”. This finding suggests that linguistic experience in digital dialect interaction is not solely governed by a single factor such as identity or emotion, but rather manifests as an integrated experiential structure in which emotional experience and social interaction perception are interwoven (Li and Shen, 2025).

Specifically, the dimension “emotional contagion and expression intensity” (F1) primarily encompasses the vividness of expression, emotional tension, and interactive appeal; the dimension “social identity and interaction inclination” (F2) reflects users' perceptions of familiarity, closeness, and propensity for social engagement during interactions. A significant positive correlation between the two dimensions indicates a high degree of consistency between emotional experience and social relationship perception in digital dialect interaction. In other words, when users perceive dialectal expressions as carrying stronger emotional tension, they are more likely to develop a sense of interactional proximity and social familiarity.

### 5.2 Emotional contagion and interactional characteristics of dialectal expression

The research findings indicate that, in comparison to Mandarin expressions, participants' positive evaluations of Xinjiang Mandarin dialect expressions were more concentrated on the “emotional contagion and expressive intensity” dimension (F1). These results suggest that dialectal expressions exhibit a greater tendency for higher evaluations concerning emotional vividness, interactive appeal, and expressive authenticity (Goldenberg and Gross, 2020; Gu et al., 2026). This implies that within digital social contexts, dialect functions not merely as a regional linguistic form but also as a communicative resource capable of enhancing the interactive atmosphere and emotional expression. This finding aligns with existing digital language research discussions on colloquial expressions, emotional transmission, and interactive styles (Al-Rawi, 2025). Prior studies have posited that language choice in online interactions is not solely determined by semantic accuracy; the linguistic form itself can influence users' understanding of the expresser's attitude, emotional state, and interactive intent (Mei, 2024). Compared to standardized language, dialects typically possess more pronounced tonal characteristics, colloquial rhythms, and contextual familiarity (Zhang and Ren, 2024), thus more readily eliciting immediate emotional responses and interactive attention. In short-video comments, instant messaging, and lightweight social expressions, users often perceive a stronger sense of “authenticity” and “presence” through dialectal forms (Wu and Liang, 2025). Consequently, in digital media environments, linguistic form itself no longer exclusively fulfills informational transmission functions (Futrell and Hahn, 2022); it has become an integral component in the formation of emotional expression, humor construction, and social atmosphere (Holmes et al., 2024). This study further demonstrates that the emotional value of digital dialect does not solely stem from linguistic variation but rather reflects a composite perceptual experience generated by the convergence of linguistic form and the digital interaction context.

### 5.3 Correlational features of social identity and interaction inclination

Beyond emotional experience, dialect expressions also exhibit distinct characteristics at the level of social interaction (Xie et al., 2023). The EFA results indicate that items such as “expression more akin to in-group communication,” “greater willingness to use such expressions,” and “easier formation of interaction” collectively constitute the dimension of “social identity and interaction inclination” (F2). This suggests that, in digital interaction, dialect expressions not only enhance emotional expressiveness but also maintain a stable association with social familiarity and interaction proximity (Choo et al., 2026; Zeier et al., 2023). However, the present study does not interpret these dimensions as mutually independent or as following a fixed sequential developmental order. Certain items exhibited moderate cross-loadings in the factor analysis, particularly those pertaining to “emotional resonance”, “expressive (infectiousness)”, and “local familiarity”, showing relatively high associations with both factors. This indicates that, within digital dialect interaction, emotional experience and social identity may not be strictly separable but rather manifest as simultaneously occurring and mutually embedded interactional experiences.

Consequently, the relationship between dialect use and willingness to interact can be elucidated through the concept of linguistic solidarity (Fernández-Mallat, 2025). Within informal digital communication, dialectal expressions can function as relational signals, indicating shared background, cultural closeness, and interpersonal openness. Dialectal use not only signifies regional identity but also fosters a sense of familiarity between interlocutors by reducing perceived social distance. This interpretation helps explain the association observed in this study between dialectal expression and a higher willingness for social connection and interaction.

### 5.4 Exploratory associations between demographic variables and dialect contact experience

Further analysis reveals weak correlations between age, dialect familiarity, and dialect usage frequency with the two experiential dimensions. Notably, the associations of dialect familiarity and dialect usage frequency with “emotional contagion and expression intensity” (F1) are relatively more pronounced. This suggests that long-term dialect contact experience may, to some extent, heighten users' emotional sensitivity and interactional receptivity toward digital dialect expressions. Meanwhile, gender differences did not reach statistical significance across either dimension, indicating no pronounced gender-based variation in the digital dialect interaction experience examined in this study. Educational attainment exhibited some differences only on the “social identity and interaction inclination” (F2) dimension; however, given the small sample sizes in certain educational subgroups and the overall limited effect, these results are best interpreted as exploratory findings and should not be overgeneralized.

### 5.5 The linguistic significance of digital dialect interaction

The findings of this study indicate that the role of dialect in social media environments has partially transcended the traditional framework of “regional identity markers” (Imuta and Spence, 2020; Zhang and Ren, 2024). For many young digital users, dialect not only serves as a regional linguistic resource but also functions as an interactive device that enhances the communicative atmosphere, heightens expressive tension, and reinforces social proximity. Consequently, dialectal practices on digital platforms exhibit both emotional transmission attributes and relational interaction attributes. Furthermore, as the corpus of this study was primarily drawn from platform-recommended content and highly interactive texts, the relevant data may be shaped to some extent by platform algorithmic visibility mechanisms (Chen et al., 2024). The higher degree of dissemination observed for certain dialectal expressions may be attributable not only to the interactional features inherent in their linguistic forms but also to platform recommendation logics, user interaction preferences, and content amplification mechanisms (McGrady et al., 2025). The findings should also be interpreted within the broader cultural and societal context of Chinese language use. Within communities on numerous social media platforms, dialects are intrinsically linked to local memory, interpersonal values, and a sense of belonging (Mei, 2024). While Standard Mandarin fulfills significant communicative and institutional functions, dialects often carry symbolic weight in informal exchanges, signifying intimacy, authenticity, and cultural heritage. Consequently, the positive perceptions of Xinjiang dialect expressions observed in this study may reflect not only linguistic preferences but also culturally embedded evaluations of familiarity and belonging within specific interactional contexts. Therefore, The current results are more appropriately understood as empirical observations within platformized digital interaction environments rather than direct equivalences to universal linguistic behaviors across all natural language scenarios.

Some degree of fluctuation persists across different stimulus materials, suggesting that the linguistic experience within digital dialect interaction is not entirely fixed but may be influenced by multiple factors, including specific expressive content, local cultural context, usage situation, and semantic familiarity. In particular, certain Xinjiang Chinese dialect expressions carry strong local cultural connotations; their Mandarin counterparts often correspond only functionally in semantic terms, making it difficult to achieve strict, full semantic equivalence. Accordingly, the “dialect–Mandarin” comparison in this study emphasizes functional communicative equivalence and proximity in overall experiential perception, rather than strict word-for-word semantic correspondence.

This study also contributes to the ongoing discourse on “standard language ideology” within digital language research (Burgers and Beukeboom, 2020; Li and Shen, 2025). For some time, Mandarin has been regarded as the more normative and efficient tool for public communication, while dialects have been viewed primarily as markers of private or localized expression. However, the present results indicate that, on digital social platforms, dialect does not diminish interactional effectiveness by virtue of its non-standard status; on the contrary, it demonstrates certain advantages in terms of emotional contagion and social proximity. This suggests that, within a platform-mediated communication environment, the interactional value of language is not determined solely by the degree of standardization, but may be more closely associated with its emotional expressiveness, interactional familiarity, and cultural presence.

### 5.6 Limitations and future research

First, the study's sample size is relatively limited and primarily drawn from Xinjiang Chinese dialect expressions within specific social media contexts. As such, the findings require further validation using larger samples, diverse regional dialect types, and varied digital platform settings. Second, this study employed exploratory factor analysis (EFA) as an initial assessment of the scale's structure. While the two empirical dimensions—“emotional contagion and expressive intensity” and “social identity and interaction tendency”—demonstrated satisfactory explanatory power, confirmatory factor analysis (CFA) was not conducted on an independent sample (Viziteu et al., 2024). Accordingly, the current results are best interpreted as exploratory insights into the empirical structure of digital dialect interaction. Third, the study adopted a cross-sectional and contextualized experimental design; the analyses primarily reflect correlational patterns among different empirical dimensions and are insufficient to support inferences of strict causal mechanisms or stage-based processes. Additionally, analyses of demographic variables were largely exploratory in nature. Although age, dialect familiarity, frequency of use, and educational level showed some associations with certain empirical dimensions, the overall effects were weak and may be influenced by sample composition and contextual factors. Meanwhile, ethnicity information was mainly used to describe the sample structure and did not yield sufficiently differentiated subgroups for stable statistical comparisons; therefore, no cross-ethnic group difference analyses were conducted. Consequently, the present findings are more appropriately applied as empirical observations within specific digital dialect interaction contexts.

With regard to qualitative analysis, this study primarily adopted a reflexive thematic analysis approach and did not employ a strict independent double-coding procedure. As a result, the thematic categorizations retain an interpretative dimension. Although the research team enhanced analytical transparency and consistency through multiple rounds of discussion, constant comparison, and verification against representative textual data, the findings may still be subject to researcher interpretation. Finally, the sample was pre-dominantly composed of young social media users aged 18–21, with a high proportion of undergraduate participants. Thus, the current results reflect language interaction experiences primarily among younger digital users and should not be directly generalized to older users, non-student populations, or the broader community of dialect speakers. Future research should incorporate more diverse sample structures, cross-regional textual data, and complex interaction scenarios to enable comparative analyses of digital dialect interaction across groups differing in age, cultural background, and linguistic experience.

## 6 Conclusion

This study investigated the expression of Chinese dialects within Xinjiang social media contexts, employing a mixed-methods approach that combined qualitative textual analysis with quantitative perceptual evaluation to examine the experiential structure and interactive features of digital dialect interactions. The results indicate that, compared with Mandarin expressions, participants generally exhibited more positive perceptual evaluations of Xinjiang Chinese dialect expressions, particularly in terms of emotional contagion, vividness, and interactional proximity. Furthermore, exploratory factor analysis revealed that participants' perceptions of digital dialect expressions did not fully correspond to the originally hypothesized independent constructs, but rather coalesced into two interrelated core experiential dimensions: “emotional contagion and expressive intensity” and “social identity and interactional orientation”. The former dimension primarily captures users' perceptions of the vividness, authenticity, and emotional tension of dialect expressions, while the latter pertains more to social familiarity, interactional closeness, and willingness to use such expressions. A significant positive correlation between these two dimensions suggests a strong association between emotional experience and social connectedness in digital dialect interaction.

The study also found that variables such as dialect familiarity, frequency of use, and age exhibited certain degrees of association with some experiential dimensions, although the overall effects were relatively limited. In contrast, gender differences did not yield significant effects. Educational attainment showed some variation on the “social identity and interactional orientation” dimension, though these findings should be interpreted as exploratory. On a theoretical level, this study further demonstrates that dialect expressions on digital platforms are no longer merely traditional markers of regional identity; rather, they have become a digital expressive resource endowed with both emotion-transmission and interaction-relationship properties. The role of dialect in social media environments is reflected not only in linguistic form but also in users' experiential perceptions of emotional expression, social intimacy, and interactional atmosphere during digital engagement. Compared with standardized language, dialect expressions are more likely to generate “authenticity” and “presence” in certain digital interaction scenarios, thereby enhancing emotional contagion and social closeness. In addition, this study shows that the experiential structure of digital dialect interaction is markedly context-dependent. Differences in stimulus materials, semantic familiarity, and cultural contexts may all influence users' perceptual evaluations of dialect expressions. Particularly within the multilingual ecological context of Xinjiang, not all dialect expressions have strict semantic equivalents in Mandarin; thus, the comparisons made here are best understood as approximations at the level of communicative function and overall experiential perception rather than word-for-word equivalence in a purely linguistic sense.

Overall, this study contributes to the understanding of emotional interaction, social identity, and non-standard language practices in digital dialect research from an experiential perception perspective, further revealing the interactional value and emotional functions of dialect on digital social platforms. The findings not only provide new empirical evidence for understanding dialect communication in digital media environments but also lay a foundation for subsequent research on the relationships among digital language, emotional communication, and social interaction.

## Data Availability

The original contributions presented in the study are included in the article/Supplementary material, further inquiries can be directed to the corresponding author.
