# Supplementary material for: The experiential structure of dialect writing in new media: a mixed-methods study combining qualitative analysis and experimental perception evaluation
Source: Front Psychol. 2026 Sep 25;17:1855166. doi: 10.3389/fpsyg.2026.1855166 (PMC13650753; doi:10.3389/fpsyg.2026.1855166)
Supplement: Supplementary file 1 [file Table_1.docx]

**Supporting Materials 1**

**Experimental Project:**

A Survey of Perceptions of Emotional State in Language Between Xinjiang Chinese Dialect and Standard Mandarin

**Experimental Requirements:**

You will read pairs of sentences, each pair containing two versions expressing the same meaning. (A) The expression in Xinjiang Chinese dialect. (B) The expression in Standard Mandarin. Please rate each sentence on a 5-point scale based on your perception, where 1 = strongly disagree, 2 = disagree, 3 = agree, 4 = mostly agree, and 5 = strongly agree.

**I. Demographic Information Collection**

Q1. Age:

Q2. Ethnicity:

Q3. Gender: Male or Female

Q4. What is your highest level of education? High school or below, Associate's/Bachelor's degree, Postgraduate.

Q5. How familiar are you with the dialect used in this study?

1=Completely unfamiliar, 2=Slightly familiar, 3=Somewhat familiar, 4=Very familiar, 5=Native speaker/Very fluent.

Q6. How frequently do you use the dialect in daily communication?

Never, Occasionally, Sometimes, Often, Very frequently.

**II. Experimental Design**

| Experiment Number | Language type | Experimental content  (The text in parentheses is an English translation.) |
| --- | --- | --- |
| Experiment 1 | Xinjiang Chinese dialect | Er Zi Wa Wa |
|  | Standard Mandarin | Nan Zi Han (Real man) |
| Experiment 2 | Xinjiang Chinese dialect | Deng buzhan |
|  | Standard Mandarin | Fang bu kai (Unable to let go) |
| Experiment 3 | Xinjiang Chinese dialect | Ai Lai Bai Lai |
|  | Standard Mandarin | Shuo San Dao Si (To complain or gossip casually) |
| Experiment 4 | Xinjiang Chinese dialect | Hai Li Mai Si |
|  | Standard Mandarin | Quan Bu (All) |
| Experiment 5 | Xinjiang Chinese dialect | Hu Ri Gui |
|  | Standard Mandarin | Hu Zheng (Reckless) |
| Experiment 6 | Xinjiang Chinese dialect | Zan Jin De Heng |
|  | Standard Mandarin | Fei Chang Hao (Very Good) |
| Experiment 7 | Xinjiang Chinese dialect | Pi Ya Zi |
|  | Standard Mandarin | Yang Cong (onion) |
| Experiment 8 | Xinjiang Chinese dialect | Lao Dao |
|  | Standard Mandarin | Li Hai, Hen Bang! (Awesome, great!) |
| Experiment 9 | Xinjiang Chinese dialect | Ou hou! |
|  | Standard Mandarin | Aa ya! (Sigh) |
| Experiment 10 | Xinjiang Chinese dialect | Za Ban Ne Sa? |
|  | Standard Mandarin | Zen Me Ban Ne? (What should we do?) |
